# Mechanism for rapid growth of organic–inorganic halide perovskite crystals

**DOI:** 10.1038/ncomms13303

**Published:** 2016-11-10

**Authors:** Pabitra K. Nayak, David T. Moore, Bernard Wenger, Simantini Nayak, Amir A. Haghighirad, Adam Fineberg, Nakita K. Noel, Obadiah G. Reid, Garry Rumbles, Philipp Kukura, Kylie A. Vincent, Henry J. Snaith

**Affiliations:** 1 Clarendon Laboratory, Department of Physics, University of Oxford, Parks Road, Oxford OX1 3PU, UK; 2 National Renewable Energy Lab, Chemistry & Nanoscience, Golden, Colorado 80401, USA; 3 Department of Chemistry, University of Oxford, Inorganic Chemistry Laboratory, South Parks Road, Oxford OX1 3QR, UK; 4 Physical and Theoretical Chemistry Laboratory, Department of Chemistry, University of Oxford, South Parks Road, Oxford OX1 3QZ, UK

## Abstract

Optoelectronic devices based on hybrid halide perovskites have shown remarkable progress to high performance. However, despite their apparent success, there remain many open questions about their intrinsic properties. Single crystals are often seen as the ideal platform for understanding the limits of crystalline materials, and recent reports of rapid, high-temperature crystallization of single crystals should enable a variety of studies. Here we explore the mechanism of this crystallization and find that it is due to reversible changes in the solution where breaking up of colloids, and a change in the solvent strength, leads to supersaturation and subsequent crystallization. We use this knowledge to demonstrate a broader range of processing parameters and show that these can lead to improved crystal quality. Our findings are therefore of central importance to enable the continued advancement of perovskite optoelectronics and to the improved reproducibility through a better understanding of factors influencing and controlling crystallization.

## Abstract (web summary)

Single crystals of lead halide perovskites exhibit good optoelectronic properties. Here, the authors study and deduce the mechanism for crystallisation and show how controlling dissolution of colloids through varying the acidity and temperature can improve the quality of the single crystals.

## Graphical abstract

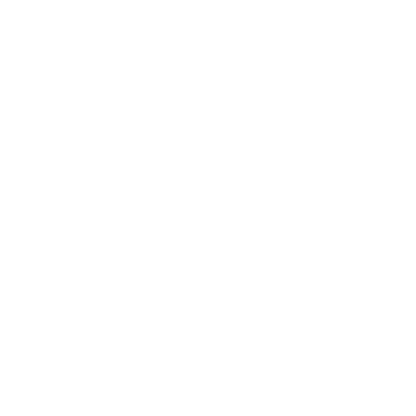

Organic–inorganic halide perovskite semiconductors, archetypically CH_3_NH_3_PbX_3_ (X=Cl, Br or I), have attracted significant attention because of their remarkable performance in optoelectronic devices^1^,^2^,^3^. A key driving factor to the optoelectronic performance is the low energy of crystal formation and the relative ease with which highly crystalline films of high electronic quality can be produced^4^,^5^,^6^. In spite of this ease of processing and remarkable performance, there remain many open questions with regards to the fundamental, intrinsic properties of this class of materials. For a crystalline material, the use of single crystals is frequently seen as the ideal platform for discerning these intrinsic properties. However, the processing pathways typically used for single crystals suffer from low throughput, which hinders their study on a broad scale. Macroscopic perovskite crystals have recently been realized by a rapid, facile crystallization route, which has been attributed to an ‘inverse solubility' effect^7^,^8^,^9^. In addition to delivering high-quality crystals^10^, the nature of the rapid crystallization is closely related to the crystallization of perovskites in thin films^11^, and proper understanding of the mechanism will enable a critically needed advance in the reproducibility and quality of both thin films and single crystals for optoelectronic devices.

The driving force for crystallization from solution is supersaturation; therefore, determining the cause of crystallization is synonymous with determining the mechanism by which a solution reaches its solubility limit. Typically, to define solubility, the saturation concentration is presented as a function of temperature. Inverse solubility, which is still presented as the temperature dependence of the saturation point, exists when the saturation concentration decreases with increasing temperature. Solubility, normal or inverse, is a thermodynamic consideration and, as such, a given salt–solvent system at a given concentration should reach saturation at the same temperature, and in the same amount of time. However, we find that the time and temperature required for crystallization of metal halide perovskites vary depending on the age of the solvent (dimethylformamide (DMF) or γ-butyrolactone (GBL)). In addition, significantly different saturation concentrations have been reported for the same salt–solvent combinations at the same temperature^7^,^9^,^12^. These observations point towards a different mechanism, beyond simple ionic solubility consideration, by which saturation is reached.

In this work, we show that the crystallization in solution is initiated by an *in situ* change in the solvent composition, specifically a change in acid–base equilibrium that raises the concentration of solute species, because of the dissolution of colloids, resulting in supersaturation. With this knowledge, we establish the rapid crystallization of macroscopic perovskite crystals across a wider range of concentrations, temperatures and solvents than previously reported and reveal how this broader parameter space can produce crystals of comparatively enhanced optoelectronic quality by enabling crystallization at lower temperatures.

## Results

### Changes in the solvent

**Figure 1:**
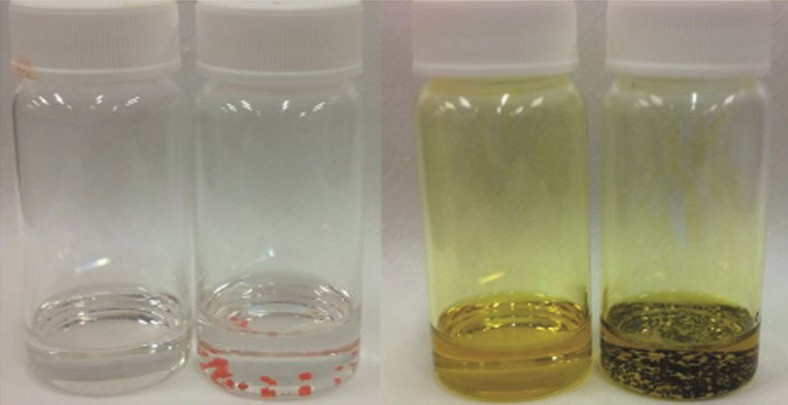
Effect of acid addition to growth solution. Photograph of the acid-initiated crystallization for the bromide (clear solution) and iodide (yellow solution) systems; the vials with no crystals were kept for 24 at the crystallization temperature with no added acid and the vial with crystals had acid added ∼2 h before the picture being taken.

We begin our investigation by looking at the solvent and how its composition may change in the current system. The reported routes to grow CH_3_NH_3_PbBr_3_ or CH_3_NH_3_PbI_3_ single crystals have predominantly involved the dissolution of the reagent salts in DMF or GBL, respectively, with a subsequent rise in temperature until crystallization occurs^7^,^8^,^9^. Because the solvents used were specific, the same for all reports, and in all but one report exclude dimethylsulfoxide (DMSO), we reviewed the typical decomposition products for DMF, GBL and DMSO. DMF can decompose into formic acid (FAH) and dimethylamine and GBL can decompose to γ-hydroxybutyric acid (GHB)^13^,^14^; in contrast, DMSO produces no acidic component upon decomposition. To determine whether this change in solvent composition (and acidity), independent of temperature, could induce the spontaneous crystallization of organic–inorganic halide perovskites, we test the crystallization in both DMF, with the addition of FAH and GBL, with the addition of GHB (the decomposition products). We begin by replicating the systems as previously reported, a 1 M solution of bromide salts (CH_3_NH_3_Br and PbBr_2_) in DMF and iodide salts (CH_3_NH_3_I and PbI_2_) in GBL, but with temperatures ∼25–30 °C lower than those required to induce crystallization in the prior report^7^. Under these conditions we do not observe any crystallization, even after several hours of heating. We then add FAH to the DMF and GHB to the GBL, and we observe that crystallization begins within 5 min. In Fig. 1 we show an image of the crystals in solution alongside the control vials into which no acid was added. Since DMSO has no acidic byproduct, we can verify whether acidity alone can cause supersaturation by attempting to crystallize the chloride system in DMSO by addition of FAH^15^. We observe that a concentrated solution of the chloride salts (CH_3_NH_3_Cl and PbCl_2_ at 2 M concentration) does not precipitate crystals when incubated at elevated temperatures (up to 80 °C) for several hours. However, on addition of FAH (3 vol% with respect to the initial solution), we observe crystals of CH_3_NH_3_PbCl_3_ being formed within 5 min at 70 °C, indicating that the effect is initiated by a change in the solvent composition and not directly related to temperature. We note that Liu *et al*.^9^ have reported crystallization of CH_3_NH_3_PbCl_3_ from DMSO at 2 M and 100 °C. We are unable to reproduce their results without the addition of FAH, but highlight this as an ambiguity. We show photographs, absorption spectra (Supplementary Figs 1 and 2) and X-ray diffraction data (Supplementary Table 1) of CH_3_NH_3_PbCl_3_, CH_3_NH_3_PbBr_3_ and CH_3_NH_3_PbI_3_ single crystals in the Supplementary Information to confirm the materials and their crystallinity.

**Figure 2:**
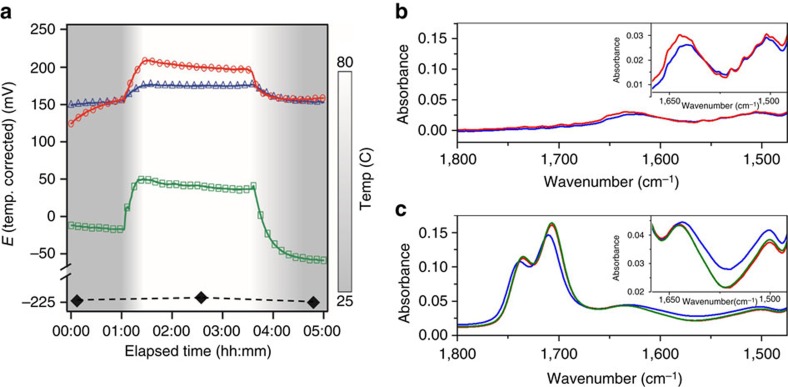
Effect of temperature on the acid–base equilibria. (**a**) Potential measurements for DMSO; neat (black diamonds), with 1 M chloride salts (green, squares), with 3 vol% FAH (red, circles) and with 1 M iodide salts and 3 vol% FAH (blue, triangles), temperature indicated by the background gradient with the scale bar to the right. (**b**) *In situ* Fourier transform infrared (FTIR) data of 1.5 M solution of chloride salts in DMSO with no FAH added and (**c**) with FAH added; data are taken initially at 25 °C (red), at 55 °C (blue) and then again after cooling back to 25 °C (green). Insets are expanded to show the MA/MAH^+^ peaks between 1,500 and 1,650 cm^−1^.

In previous reports the crystallization was reversible, that is, when the temperature was decreased, crystals were dissolved; although the addition of an acid is not reversible (nor is the *in situ* production of acid from the solvent due to solvent degradation), the change in acidity (protonation or deprotonation of the acid) can be reversed. To verify that this change in acidity can occur, we perform electrochemical experiments and record the potential change of a pH-specific electrode as a function of temperature for a variety of systems. We utilize DMSO, because of its lack of acid byproduct, to test solutions with the reagent salts only, with 3 vol% added FAH only, and with both the salts and the FAH (we performed similar test for DMF, GBL and the neat solvents and show details in Supplementary Figs 3 and 4). We show the key results in Fig. 2a; for all solvents the addition of only the reagent salts (CH_3_NH_3_X+PbX_2_) results in an increase in proton activity before any heating. Given that the only acid component in the DMSO solution is CH_3_NH_3_^+^ (MAH^+^), this must be due to the deprotonation of MAH^+^ to methylamine (MA) and H^+^. In addition, for all solvents with both salts and added acid, the acidity is not additive for the salts and acid alone, which points to a shift in the acid/base equilibria as we depict below:


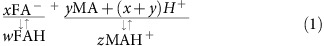


where *w*, *x*, *y* and *z* are undetermined stoichiometric coefficients. It is also clear that, when all components are present, the change in acidity is reversible. We note that these data infer that the change to the solvent with increasing and reducing temperature is not the production of additional acid by solvent degradation, but a shift in the equilibria in equation (1).

To determine which species are changing, we perform *in situ* attenuated total reflection infrared (ATR-IR) spectroscopy of solutions at different temperatures. There are two peaks of interest for both of the acids present; for FAH they are nominally at 1,700–1,750 cm^−1^ and for MAH^+^ at 1,500 and 1,625 cm^−1^ ; for both acids the absorbance should decrease as the acid is deprotonated (see Supplementary Discussion)^16^,^17^. In Fig. 2b,c, we show the infrared absorption spectrum of 1.5 M CH_3_NH_3_Cl+PbCl_2_ in DMSO initially at 25, at 55 and again at 25 °C after cooling. In Fig. 2b we show the result with no FAH added; we observe a slight decrease in the absorbance, consistent with the deprotonation of MAH^+^ at elevated temperature, which correlates with the electrochemical results. In Fig. 2c we show the result when FAH is included. Here we observe that, in the presence of both acids, the increase in temperature causes the deprotonation of FAH (absorbance decreases) and the protonation of MA (absorbance increases); we observe that when we cool, the effect is reversible (control experiments were performed and the results shown in Supplementary Figs 5–7 and discussed in the Supplementary Discussion). We interpret these ATR-IR results to indicate that the heating of a solution of the reagent salts in a solvent that includes a weak carboxylic acid pushes the MA/MAH^+^ equilibrium in equation 1 towards increasing MAH^+^.

The combination of the electrochemical and ATR-IR results allows us to determine that the solvent, which we typically take to be, for example, DMF, is more accurately described as DMF+FAH+MA, where the FAH can come from the natural decomposition of DMF or from direct addition. The change in the solvent that influences the supersaturation concentration of reagent salts is when FAH deprotonates, causing a reduction in MA; this change in FAH can be affected by raising the temperature; however, it can also be initiated by adding FAH isothermally.

Determination of the changes to the solvent, specifically the existence and change in concentration of MA, prompts us to consider the role MA plays in solvation. Previous reports have shown a strong solvent-annealing effect with MA vapour and that a solution of CH_3_NH_3_PbI_3_ can be made in MA at 85 wt% (ref. 18). When we bubble MA into a vial containing CH_3_NH_3_PbBr_3_ crystals, in equilibrium with a saturated solution of the bromide salts, the crystals dissolve implying that the MA/solvent mixture acts as a stronger solvent than the neat solvent alone (See Supplementary Discussion for details). The summary of our experiments exploring the solvent is that it changes by a change in acidity and that the solvent becomes weaker as the acidity is increased.

### Changes to the solutes

**Figure 3:**
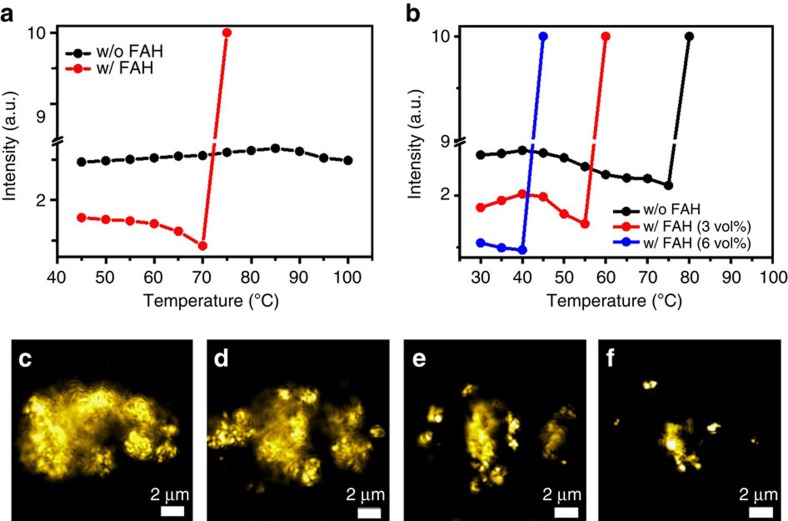
Colloid dissolution effect. (**a**) Effect of added acid and temperature on the scattered light intensity of CH_3_NH_3_Cl+PbCl_2_ in DMSO. (**b**) Effect of added acid and temperature on scattered light intensity of CH_3_NH_3_Br+PbBr_2_ in DMF. In both the graphs, the value of 10 (a.u.) represents the saturation point of the detector. (**c**–**f**) Micrographs from iSCAT imaging of dissolution of colloids after addition of FAH (10 vol%) in the 1 M CH_3_NH_3_I+PbI_2_ in GBL at room temperature (see Supplementary Movie 1).

We next use our knowledge of the solvent changes to consider how it affects the solutes. Previous work has shown that these solutions consist not only of solvated ions but also contain lead halide colloids and complexes. To determine whether the colloids have an impact on the crystal formation, we remove some fraction of the colloids by centrifuging the salt solutions where we expect the solvated ionic species to remain in the solution because of their smaller size. We see that removing the colloids dramatically changes the onset temperature of crystallization as well as the yield (Supplementary Fig. 8 and Supplementary Discussion). This indicates that the ions that are incorporated in the colloids at room temperature are important for and involved in some manner in the perovskite crystallization. If the colloids dissolve with an increasing temperature, then this could be the source of ions that lead to supersaturation. In order to assess whether increasing temperature alone can result in dissolution of the colloids, we perform static light-scattering (SLS) experiments as a function of temperature; generally, a decrease in the scattering intensity denotes a decrease in the mean colloid particle size and/or particle concentration. We present results for a solution of 2 M chloride salts in DMSO (no acidic degradation product from the solvent; the only acid present is MAH^+^) in Fig. 3a, and we observe a negligible change in scattering intensity with an increasing temperature. This indicates that temperature, and the small resulting change in acidity shown in Fig. 2a (black diamonds), has a negligible influence upon the colloids in this system. Upon the addition of FAH (2.5 vol%) to the 2 M chloride salts in DMSO, we observe a monotonic decrease in the light-scattering intensity as the solution is heated. This decrease in scattering intensity continues until ∼75 °C where we see a sharp rise in scattering intensity. Visual inspection at this temperature reveals that this inflection coincides with the formation of perovskite crystals. This is a clear indication that an increase in acidity can cause dissolution of colloids and subsequent rapid crystallization of perovskites. In Fig. 3b, we show light-scattering data for the bromide salts in DMF, where we also observe a decrease in scattering intensity as the temperature is increased, even without added FAH. For this system, this may be because of some small amount of FAH being present in fresh DMF or a stronger deprotonation of MAH^+^ than in DMSO, or the colloids are more labile in this system. However, with an increasing concentration of FAH in the bromide salts in DMF solution, we observe the light-scattering intensity drop further, and the onset temperature for crystallization (as indicated by inflection of the scattering) drops monotonically to lower temperatures. We observe similar results for the iodide salts in GBL (Supplementary Fig. 9). To summarize this section, the light-scattering results are consistent with the colloids being increasingly dissolved or broken up with an increasing acidity (where the acidity is a function of temperature), until the point at which supersaturation is reached and the crystallization proceeds. We also note that we observe an increase in the light-scattering intensity upon lowering the temperature, which finally returns to the initial value, which is consistent with the dissolution of and formation of colloids being reversible. We associate this reversible dissolution to the reversibility of proton activity in the solution that, in turn, depends on the temperature of the system.

In order to confirm and visualize the dissolution of the colloids, we employ interferometric scattering microscopy (iSCAT) and show micrographs of a time sequence of the iodide salts dissolved in GBL following the addition of FAH in Fig. 3c–f (see Supplementary Movie 1). We observe the colloids breaking up and dissolving with the addition of FAH, even at a fixed temperature, confirming our interpretations from the SLS. We show the iSCAT images as a function of increasing temperature in Supplementary Fig. 10 and confirm that the decrease in intensity in the light-scattering data corresponds to a disintegration of aggregated colloids followed by dissolution of the colloids. Importantly, during these measurements, we captured images of crystallization occurring. In Supplementary Fig. 11 we show a sequence of events during MAPbBr_3_ crystal formation where we see that the crystals are growing from clear solution rather than from detectable colloids (see Supplementary Movie 2). This indicates that the visible colloids are an important source of free ions, but are not directly partaking in crystal growth. In summary of our examination of the solute, the colloids are in equilibrium with solvated ionic species and an increase in acidity dissolves the colloids in solution, leading to an increased concentration of dissociated ions.

## Discussion

**Figure 4:**
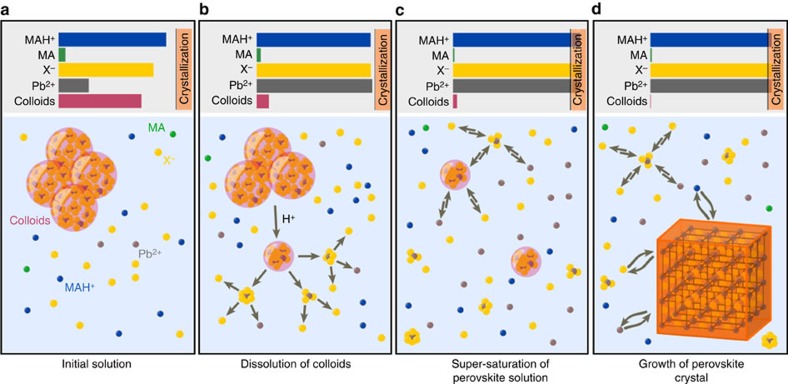
Schematic representation of the crystallization mechanism. Horizontal bars on the top of each panel represent the concentration Pb^2+^, X^−^, MAH^+^, MA and colloids (not to scale), and spheres with similar colour schematically represent each species in the bottom panel. The vertical bar in the top panel represents the regime for crystallization (that is, supersaturation). (**a–d**) show the sequence for the crystal growth.

Our examination of both the solvent and the solutes shows that both are changing as the acidity of the solution is increased. Further, we show that this increase in acidity can be initiated by the direct addition of acid, and, although a change in temperature will also increase the acidity, it is not a necessary condition. From our solvent examination we know that an increase in acidity lowers the strength of the solvent by protonating MA and removing it from the solvent (MAH^+^ is a solute). From our solute studies we know that the increase in acidity dissolves the lead halide colloids. This allows us to hypothesize the mechanism for crystallization, which we visualize in Fig. 4 where we schematically represent the state of the system at several key points. In its initial state, the solution is depleted in all the components, that is, the Pb^2+^ and halide ions contained in colloids, and the MAH^+^ because of partial deprotonation. When proton activity increases by deliberate addition to the system or by dissociation of existing acid with increasing temperatures, the following three changes occur: first, lead halide colloids are dissolved and they produce a higher concentration of the ion species required for crystal formation; second, the concentration of MAH^+^ is increased because of protonation of the MA; and, third, the solvent becomes weaker because of a lower concentration of the MA. In other words, an increase in acidity raises the concentration of all solutes, while simultaneously decreasing the strength of the solvent; this results in supersaturation and the onset of crystallization. We tested our hypothesis through a variety of controls including shifting the acid/base equilibria via addition of different bases (Supplementary Fig. 12) and isothermal crystallization of all systems discussed; we give details and discussion of these controls in the Supplementary Discussion. We note that we can also crystallize the caesium system (CsPbBr_3_) by addition of acid to a DMSO solution (details in the Supplementary Discussion, Supplementary Figs 13 and 14 and Supplementary Table 2); although this does not discount the change in solvent strength in those systems where MAH^+^ exists, it does infer that the effect of dissolving the colloids can be sufficient by itself to induce supersaturation and crystallization.

During revisions of this manuscript there has been a report of crystallization of MAPbCl_3_ using DMSO, but with the addition of chlorobenzene (CB) to the solution, which is an antisolvent for the perovskite^19^. This report indicates that the addition of an antisolvent is also capable of initiating crystallization, and of reducing the onset temperature for crystallization. This raises the question as to whether the antisolvent is simply reducing the solvent strength, or also influencing the dissolution of colloids, whether directly, or through an indirect influence upon the acid–base equilibrium of the solution. In addition, it raises the question as to whether or not FAH is simply acting as an antisolvent. In order to confirm that FAH is not simply an antisolvent, we attempted to dissolve CH_3_NH_3_PbI_3_ perovskite crystals in FAH and CB selectively. While for CB we observe that the perovskite crystals remain undissolved, for FAH, they dissolve into a turbid solution (see Supplementary Fig. 15). This indicates that FAH is not a typical antisolvent. There clearly remain open questions as to the relationship between our observed acid-induced crystallization and the antisolvent-induced crystallization. Specifically, a deeper level of knowledge is still required to understand what fundamentally drives the dissolution of colloids, which shifts the concentration of free ions into the supersaturated regime. Understanding these further aspects is the focus of ongoing research in our groups.

It is commonly understood that single crystals of semiconducting materials ultimately have improved properties over polycrystalline thin films because of the elimination of grain boundaries and compositional inhomogeneity. Hence, the understanding of single-crystal growth we have delivered here will have a significant impact upon the community advancing single-crystal perovskites for optoelectronics. Here we demonstrate three accomplishments enabled by this deeper knowledge including crystallization of additional salt/solvent combinations and an increased yield (see Supplementary Discussion). We also demonstrate the preparation of crystals of all three salts over a wider range of temperatures, ranging from 50 to 80 °C for the chloride, 30 to 100 °C for the bromide and 48 to 120 °C for the iodide systems (see Supplementary Table 3 and Supplementary Fig. 16). In Supplementary Fig. 1 we show the optical images of centimetre-scale crystals of CH_3_NH_3_PbCl_3_, CH_3_NH_3_PbBr_3_ and CH_3_NH_3_PbI_3_ grown at 55 °C. Further, we demonstrate the impact of this broadened set of processing parameters by growing iodide crystals below the tetragonal–cubic phase transition temperature.

We can now grow CH_3_NH_3_PbI_3_ crystals at temperatures ranging from 48 to 100 °C, and we characterize them by single-crystal diffraction and optical measurements. We perform single-crystal diffraction on three crystals grown at 55, 70 and 100 °C, and show the full data set in the Supplementary Table 4. As the growth temperature decreases, we observe an improvement in the overall fit parameters, less twinning and an increased tilt between adjacent lead halide octahedra (see Supplementary Fig. 17), indicating that controlling the temperature of growth has a structural impact upon the final crystal.

**Figure 5:**
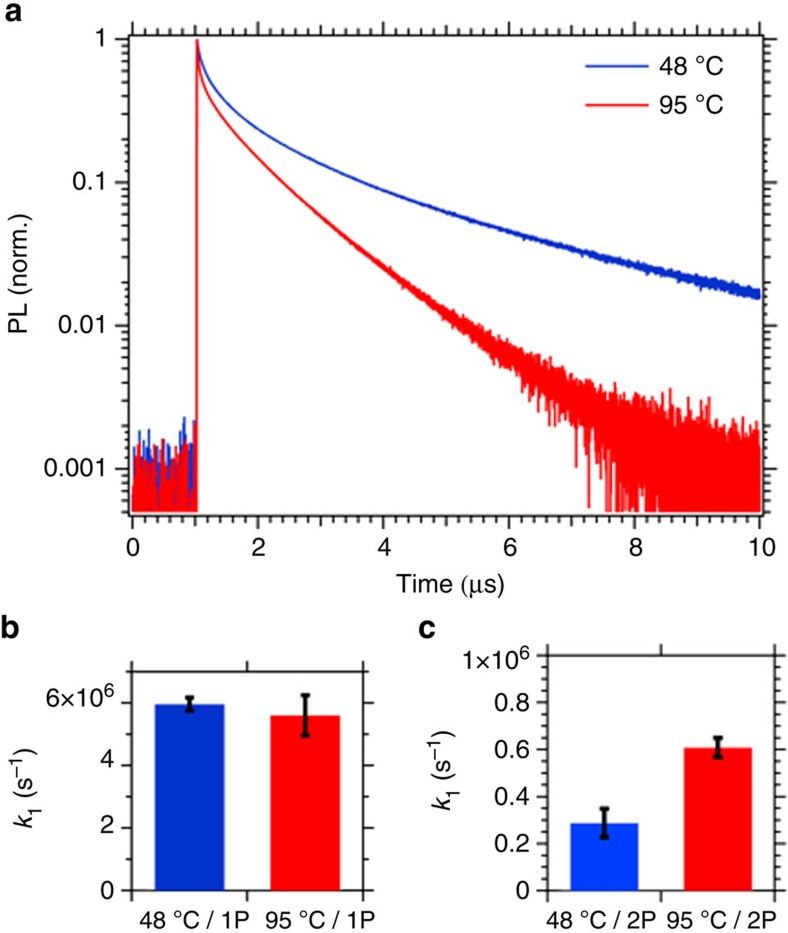
Photoluminescence of CH_3_NH_3_PbI_3_ single crystals grown at 48 °C (below the tetragonal to cubic phase transition) and at 95 °C. (**a**) 2P excitation PL transient (*λ*_ex_=1,400 nm, fluence=37 mJ cm^−2^); (**b**,**c**) first-order recombination coefficients for 1P and 2P excitation. The recombination coefficients are averaged over five different excitation fluences (1P excitation at 532 nm: 14.3, 30.9, 54.5, 73.3 and 131.8 μJ cm^−2^; 2P excitation at 1,400 nm: 9.0, 12.8, 13.2, 17.4 and 26.6 mJ cm^−2^, see Supplementary Fig. 19). The errors bars show the s.d.

We next explore the impact of the temperature of crystallization upon the optical properties of crystals. We show time-resolved photoluminescence (PL), using both one-photon (1P) and two-photon (2P) excitation, on crystals grown at 48 °C (below the tetragonal to cubic phase transition) and 95 °C in Fig. 5a (2P) and Supplementary Fig. 18 (1P). Photoluminescence in lead halide perovskites originates from band-to-band recombination of free electrons and holes^20^. The second-order recombination rate scales as the product of the electron and hole number density. However, at low excitation fluences, the lead halide perovskites exhibit a first-order recombination rate, governed by trap-assisted recombination. For 1P excitation (2.33 eV), the incident light of above band gap energy (∼1.55 eV) is absorbed strongly in the crystal within a penetration depth of ∼170 nm. Hence, most of the free charges are generated near the surface of the crystal. For 2P excitation, the sub-band gap photon energy (0.89 eV) has very low absorbance; owing to this, the light absorption, and charge generation, is distributed over the entire thickness of the crystal. As a result, the 2P measurements probe a substantially larger bulk volume than surface volume, and the 2P PL data contain more information about the bulk properties^21^. For both 1P and 2P PL, the initial decay is dominated by a fast process, followed by a slower, exponentially decaying component. We quantify the slow first-order decay rates by fitting the slowest part of the decays measured at various excitation fluences with a single exponential. With 1P excitation, the first-order decay coefficient, *k*_1_, is equivalent for crystals grown at different temperatures. We note that the estimated *k*_1_ (Fig. 5b) is very similar to high-quality polycrystalline perovskite films used in photovoltaic devices (*k*_1_∼5 × 10^6^ s^−1^; ref. 20). This implies that the surface region of single crystals is similar to polycrystalline thin films in terms of recombination site density. As we expect, the 2P measurements reveal longer PL lifetimes for both crystals, with *k*_1_ (Fig. 5c) reduced by one order of magnitude, in agreement with the values found by Yamada *et al*.^21^. Because of these long lifetimes we can infer that the bulk defect density in single crystals is lower than that at the surface. When the crystal is grown at 48 °C, the recombination is further decreased by a factor of 2 with respect to the 95 °C grown crystal. Thus, our measurements are consistent with the crystal grown at lower temperature having a lower non-radiative recombination site density in the bulk of the crystal, and hence improved optoelectronic quality.

In summary, we have presented compelling evidence that the observed rapid crystallization of metal halide perovskite crystals is because of the dissolution of colloids that can happen by a change in acidity of the solution, and because of a change in the solvent strength. The dissolved colloids increase the concentration of free ions in solution, leading to supersaturation and the onset of crystallization. Our findings open a whole new depth of questions into understanding the interaction between solvent and colloids, which will catalyse further investigations. By direct addition of formic acid, we have demonstrated a much broader range of parameters including the temperature range and the solvent/salt combinations. By decoupling the temperature from the acidity, we have shown an increased reaction yield, the growth of additional crystal systems and the growth of higher-quality crystals with improved photoluminescence properties. High-quality crystalline perovskites, in both large crystals and thin films, are at the core of the phenomenal optoelectronic properties of these materials. The thin film deposition is typically from the same solvents as used for the single-crystal growth, and the same acid byproducts will be present, at currently uncontrolled concentrations. Our work here will, therefore, have a direct impact upon researchers working on polycrystalline thin films, in addition to single crystals. The findings we report here are therefore of central importance in enabling the continued advancement of perovskite optoelectronics and in the improved reproducibility, homogeneity and eventual manufacturability of these technologies.

## Methods

### Materials

We bought DMSO, DMF, N-methyl-2-pyrrolidone (NMP), carboxylic acids, PbI_2,_ PbBr_2_ and PbCl_2_ from Sigma-Aldrich, GBL from Alfa Aesar, MAHI and MAHBr from Dyesol, and MAHCl from Merk. We used the chemicals as received without further purification.

### Synthesis of GHB

A volume of 20 ml of fresh, neat GBL was added dropwise to 15 ml of 0.02 M NaOH solution while stirring; a precipitate forms immediately. After complete addition, the solution was stirred for 30 min and the precipitate, NaGHB salt, recovered using a rotovap. The salt was then dissolved in ∼15 ml of 1.2 M HCl in water; the precipitate, NaCl, was dried in a rotovap and the liquid recovered. The recovered liquid consisted of GHB, water and unreacted HCl. The final recovery was made by boiling off the remaining water/HCl at 60 °C for 48 h. The distillation was taken to be complete when the remaining liquid formed a very viscous liquid and the pH, measured in water, matched the calculated pH of pure GHB.

### Preparation of CH_3_NH_3_PbBr_3_ crystals in NMP

We prepared 1 M solution of the bromide salts (MAHBr+PbBr_2_) in NMP and added 60 μl of FAH per 1 ml of NMP. To grow single crystals of CH_3_NH_3_PbBr_3_, we kept the resulting solution in a closed cap vial and incubated at 80 °C. Under similar conditions, solution without FAH did not yield any crystals.

### Preparation of CH_3_NH_3_PbBr_3_ crystals in DMSO

We prepared 1.5 M bromide salt solution in DMSO and added 400 μl of FAH per 1 ml DMSO. We placed the resulting solution in a vial and placed in an oil bath at 105 °C and noticed crystallization within 5 min of incubation. A control vial without FAH did not produce any crystals even after several hours of incubation.

### Preparation of centimetre-scale CH_3_NH_3_PbCl_3_ crystal at 55 °C in DMSO

We prepared 2 M solution of the chloride salts in DMSO and heated it at 80 °C with constant stirring for 2 h to get a clear solution and then cooled it down to room temperature. We then used 5 ml of the solution and added 200 μl of FAH to it and filtered the solution using a 0.45 μ filter. We kept the solution in a closed cap vial at 55 °C to produce seed crystals. We then prepared another solution—this time 150 μl of FAH per 5 ml of the salt solution. In a 30 ml vial, we put a cleaned Si wafer at the bottom of the vial, poured the solution into the vial and carefully put a seed (∼500 μm scale) crystal of CH_3_NH_3_PbCl_3_ on the Si substrate. We then closed the cap of the vial and put that in an oil bath kept at 55 °C. The crystals grew on the Si substrate, and we collected the crystal when they reached the desired size. When we wanted bigger-size crystals, we added fresh solutions to the vial and allowed the crystals to grow further.

### Preparation of centimetre-scale CH_3_NH_3_PbBr_3_ crystal at 55 °C in DMF

We prepared 1 M solution of the bromide salts in 5 ml DMF and then added 150 μl of FAH to the solution followed by a filtration with a 0.45 μ filter. We used a 30 ml vial and put a cleaned Si wafer at the bottom of the vial. We then poured the solution into the vial and carefully put a seed (∼500 μm scale) crystal of CH_3_NH_3_PbBr_3_ on the Si substrate. We follow the same procedure for crystal growth that we described for the chloride system. We note here that the amount of FAH needed to grow the crystals at 55 °C may vary depending on the existing FAH in the DMF as a degradation product.

### Preparation of centimetre-scale CH_3_NH_3_PbI_3_ crystal at 55 °C

We prepared iodide salt solution at 1.2 M in 5 ml fresh GBL by dissolving the salts at 55 °C with vigorous stirring for at least 30 min. The growth of the initial seeds and the subsequent 1 cm crystal was as described above for the chloride crystals, except that the FAH volume added was 14.5 vol%. Crystals were grown at 50, 70 and 100 °C by adjusting the molarity to 1.3, 1.0 and 0.8 M, respectively, and adjusting the volume % of FAH to 16.0, 10.0 and 7.0 vol%, respectively.

### Absorbance

We collected the absorption spectra using a Perkin-Elmer Lambda 1050 UV-Vis Spectrophotometer in the transmission mode.

### X-ray single-crystal diffraction and analysis

We collected the single-crystal data at 293 K using an Agilent Supernova diffractometer that uses Mo *K*_α_ beam with *λ*=0.71073 Å and is fitted with an Atlas detector. We did the data integration and cell refinement using the CrysAlis Pro Software, analysed the structure by Patterson and Direct methods and refined using the SHELXL 2014 software package.

### *In situ* electrochemical measurements

For electrochemical measurements, we used a Mettler Toledo SevenEasy pH meter in the potential mode using an InLab Micro glass probe. We prepared samples in 22 ml vials, with the probe inserted through a septum cap and measured on a hotplate with constant stirring; we used equivalent vials of solvent only to monitor temperature with a temperature probe. We recorded both temperature and potential in 1 s increments.

We measured each sample at room temperature (RT) for a period sufficient to stabilize the system, typically 30–45 min. Then, we raised the temperature to ∼80 °C for 30–45 min. After that, we turned off the heater and allowed the system to cool back to RT. We calibrated the pH probe before and after each measurement with a three point buffer set. Whenever we saw a difference in the offset >10 mV, we cleaned the probe thoroughly, changed the internal electrolyte and repeated the measurement (for reference at 20 °C, in water, the potential change is ∼59 mV per pH). Before the use of a new solvent, we cleaned the probe and changed the electrolyte and then calibrated the probe.

For temperature correction we used the following equation


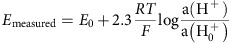


where *E*_measured_ is the recorded potential, *E*_0_ is the temperature-corrected potential of the probe, *T* is the temperature, *R* and *F* are the gas and Faraday constant, respectively, a(H^+^) is the proton activity of the sample and a(H_0_^+^) is the proton activity of the internal probe standard. We experimentally measured the value for *E*_0_ using a buffer of pH 7.0 over the temperature range of interest.

For neat, pure solvents, a constant *in situ* measurement was not possible with the experimental apparatus used because of the lack of any salt or an appreciable concentration of acid in the system. Therefore, the potential change for the pure, neat solvents (black diamonds in Fig. 2a and Supplementary Fig. 3) were taken by recording three separate measurements for each temperature of interest; measurements were recorded during 60 s after a 60 s stabilization time and the values for all three measurements averaged. In the case of DMF and GBL, these measurements were performed during the same experimental session and, using the same solvent, as the samples plotted in the accompanying plots.

### *In situ* ATR-IR spectroscopy

*Instrument details.* We used an Agilent Fourier transform infrared spectrometer with liquid nitrogen-cooled Mercury Cadmium Telluride detector for *in situ* infrared spectroscopy. For multiple reflections of incident infrared light, we used a modified ATR accessory (GladiATR, PIKE Technologies) placed inside the spectrometer.

*ATR crystal and infrared cell.* We used a Si trapezoidal ATR reflection element with a dimension of 5 × 8 × 1 mm^3^ and 39° angle of incidence at both short edges (Crystal GmbH, Germany). Before each experiment, the Si crystal was cleaned with isopropanol followed by deionized water. The infrared cell was developed in-house for *in situ* experiments^22^. The Si crystal was attached to the baseplate of the infrared cell with epoxy glue to prevent leakage during solution flow through the cell. A glass vial fitted with a rubber septum, placed in a copper heating block, was used as a solvent reservoir. To circulate the salt solution between the reservoir and the infrared cell, we used a peristaltic pump (flow speed: 5 ml min^−1^) fitted with thermally insulated tubing. The schematic arrangement is shown in Supplementary Fig. 20. To increase the temperature of the solution, we heated the copper block and monitored the temperature of the solution in the cell by insertion of a thermocouple. Spectra were recorded at 5 °C intervals. Infrared data are presented as absorbance spectra with reference spectra collected at a bare Si ATR reflection element. Spectra were collected at a spectral resolution of 4 cm^−1^ and presented as 250 co-added scans.

### Interferometric scattering microscopy

We used a custom-built iSCAT microscope^23^,^24^ to follow the colloidal aggregate dynamics and perovskite crystal growth. The microscope set-up is as follows. A 635 nm diode laser (Lasertack) serves as the light source with a P-polarization output. The collimated laser beam passes through two acousto-optic deflectors (Gooch & Housego), rapidly scanning the beam in two dimensions. A 4*f* telecentric lens system images the deflection generated by the acousto-optic deflectors into the back focal plane of a high-numerical aperture (NA) microscope objective (Olympus PlanApo, × 60, 1.42 NA). Before entering the objective, the beam passes through a polarizing beam splitter to allow for separation of the illumination and detection channels, and a quarter waveplate. A 45-degree mirror couples the illumination light into the objective. The objective collects the light reflected from the glass/sample interface together with the back-scattered light from the sample. After a second pass through the quarter waveplate, the light becomes s-polarized, and the polarizing beam splitter reflects scattered and reflected light into the detection channel. A 1 m-focal length achromatic doublet (Thorlabs) forms an image on a CMOS camera (Photonfocus), with a magnification of × 333 and a nanometre to pixel conversation of 31.8 nm px^−1^.

We cleaned the coverglass thoroughly with ultrapure water (Milli-Q) and ethanol. We then incubated the coverglass in a 50:50 mixture of isopropanol and ultrapure water for 15 min in a sonicator bath. After a final rinse with ultrapure water, we blow-dried the coverslips with nitrogen. We then placed a clean 20 μl volume silicone gasket (Grace Bio-Labs) firmly on top of the clean coverglass and then the solution to be imaged within. We used a flexible polyimide foil heater (Thorlabs) wrapped around the microscope objective and temperature controller (Thorlabs) to control the sample heating during imaging.

We captured images at 10 Hz. The exposure time for colloidal dissolution and crystal growth were 7 and 0.01 ms, respectively.

### Static light scattering

*Instrument details*. A quartz cuvette, placed on a temperature-controlled stage with a magnetic stirrer, serves as the container for the salt solutions. A 4.5 mW, 532 nm CW laser (Thorlabs CPS532) acts as the light source, which is measured using a Si diode (Thorlabs det36/a) positioned at the right angle to the light beam and in front of one of the walls of the cuvette. An optical chopper modulates the incident laser beam, and a lock-in amplifier (Stanford Research Systems SR810) acquires the data from the Si diode.

We prepared 1 M solution of MAHI+PbI_2_ in GBL, 1 M solution of MAHBr+PbBr_2_ in DMF and 2 M solution of MAHCl+PbCl_2_ in DMSO. We stirred the stock solutions with a magnetic stirrer at 50 °C for few hours before bringing them to the room temperature. We used 2 ml of the solution for the scattering experiment.

We raised the temperature to 5 °C step size and waited for 2 min at each point before collecting the intensity of the scattered light. The rotation speed of the stirrer in the cuvette remained constant during the entire experiment. For MAHCL+PbCL_2_ in DMSO, we heated the solution up to 70 °C and then brought it back to 25 °C and measured the scattered light intensity for 2 h, which was stable over the time range. In the MAHBr+PbBr_2_ in the DMF system, after the formation of crystals, the solution was brought back to 10 °C and kept there for a rapid dissolution of crystals, and then the temperature was raised for additional measurements.

### 1P and 2P photoluminescence

The 1P photoluminescence spectrum was recorded in air using 532 nm excitation and off-axis front face detection geometry in a commercial spectrometer (Horiba Fluorlog) equipped with a nitrogen-cooled charge-coupled device detector. Various sample angles between 0 and 15 degrees off-normal from the incident beam were tried, verifying that the spectra recorded were not influenced by small variations in the angle of incidence.

2P photoluminescence measurements were carried out in a sealed, nitrogen-filled cell with fused quartz windows. Photoexcitation was provided by a Nd:YAG pumped optical parametric oscillator (OPO) (Spectra Physics Quanta Ray and GWU Premi Scan) with a pulse width of 5.6 ns, full-width at half-maximum. Intensity was controlled using a set of neutral-density filters downstream of the OPO and was measured with a pyroelectric power meter (Coherent USB-10MB-LE). The laser light was focused with a 500 mm focal length fused quartz lens to a spot size of ∼0.02 cm^2^ (wavelength-dependent). The long focal length ensures little change in spot size as the excitation beam passes through the sample, and thus provides a uniform probe of the bulk of the sample without artefacts from a localized focal volume.

Photoluminescence was detected in off-axis front-face geometry. The PL detection system consists of a 50 mm *f*/0.95 C-mount camera lens (Thorlabs MVL50HS) mounted on a filter holder (Thorlabs CFH2) with a silicon avalanche photodiode (APD) (Thorlabs APD430A) attached to the other side, transducing transient optical signals with DC-400 MHz bandwidth. An 800±10 nm bandpass filter (Thorlabs FB800-10) was used to block excitation light, and to pass a reasonable amount of both observed emission bands from the sample. The whole assembly is mounted to a pitch-yaw kinematic stage for easy alignment with the emission spot. The signal from the APD was digitized using a fast oscilloscope (Tektronix DPO7254), and successive pulses were averaged in a software to obtain clean transient signals and the associated s.d.

### Data availability

The data that support the findings of this study are available from the corresponding author upon request.

## Additional information

**How to cite this article:** Nayak, P. K. *et al*. Mechanism for rapid growth of organic–inorganic halide perovskite crystals. *Nat. Commun.* **7,** 13303 doi: 10.1038/ncomms13303 (2016).

**Publisher's note:** Springer Nature remains neutral with regard to jurisdictional claims in published maps and institutional affiliations.

## Supplementary Material

Supplementary Information Supplementary Figures 1-20, Supplementary Tables 1-4, Supplementary Discussion and Supplementary References.

Supplementary Movie 1 Dissolution of colloids due to the addition of acid.

Supplementary Movie 2 Formation of crystals.
