# Supplementary material for: Mechanism for rapid growth of organic–inorganic halide perovskite crystals
Source: Nat Commun. 2016 Nov 10;7:13303. doi: 10.1038/ncomms13303 (PMC5109546; doi:10.1038/ncomms13303)
Supplement: Supplementary Information — Supplementary Figures 1-20, Supplementary Tables 1-4, Supplementary Discussion and Supplementary References. [file ncomms13303-s1.pdf]

Supplementary Information

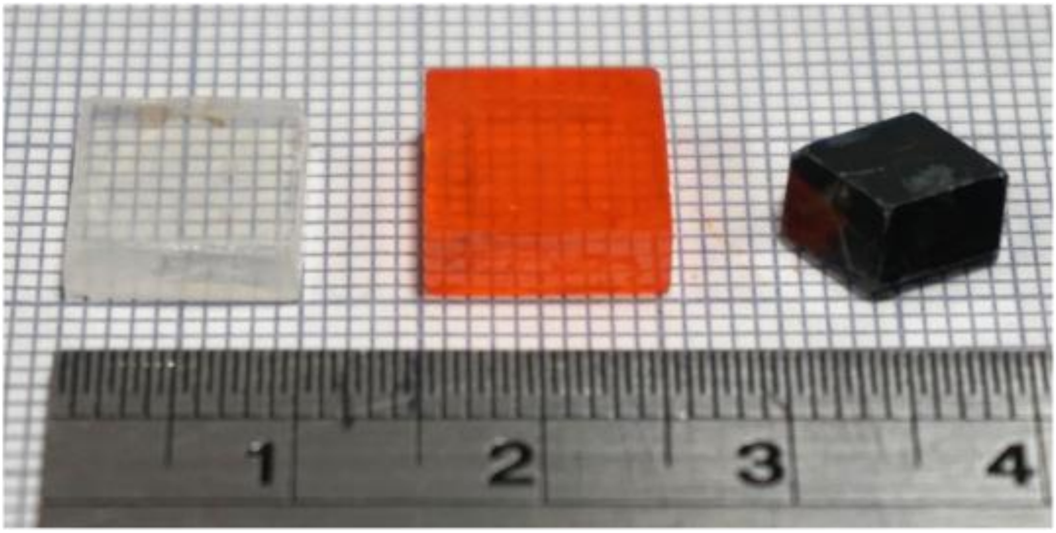

Supplementary Figure 1| Crystal Pictures. Optical image of cm scale crystals of $MAHPbCl_3$, $MAHPbBr_3$ and $MAHPbI_3$ grown at 55 °C

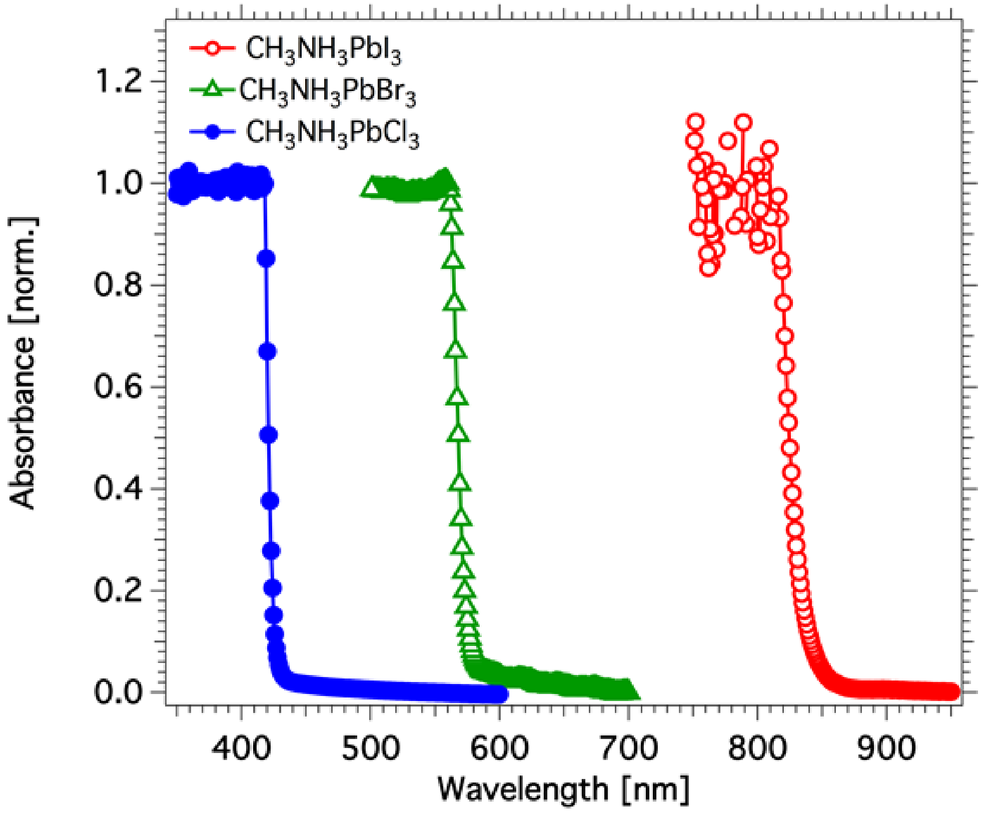

Supplementary Figure 2| Absorption Spectra. Normalized absorption spectra for $CH_3NH_3PbCl_3$ (blue, filled circles), $CH_3NH_3PbBr_3$ (green, open triangles), and $CH_3NH_3PbI_3$ (red, open circles)

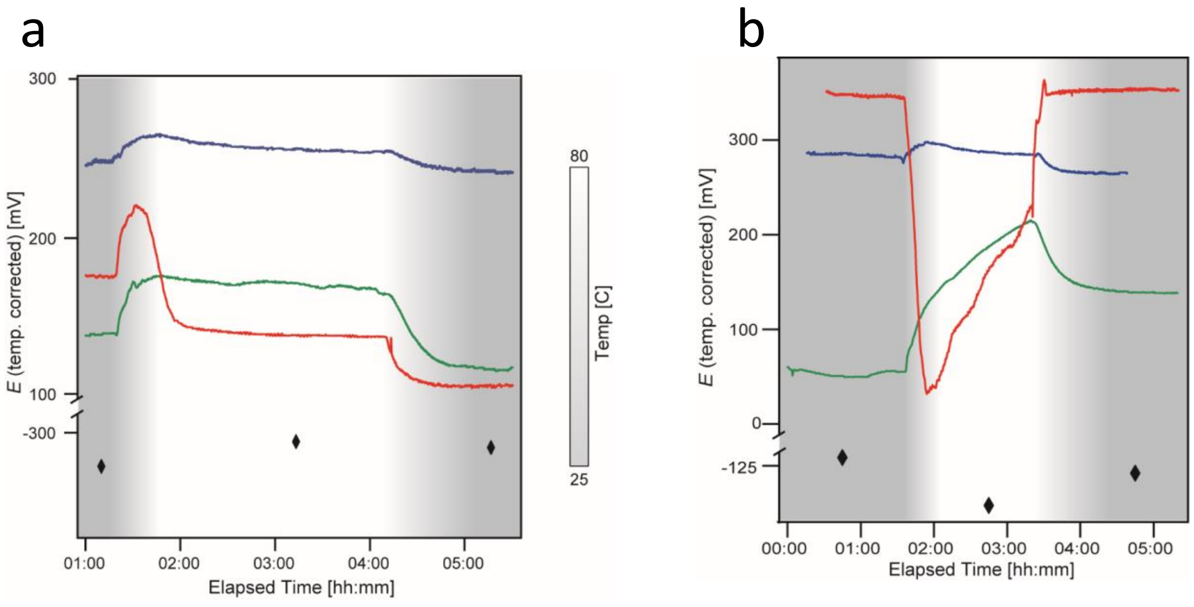

Supplementary Figure 3| Electrochemical data with salts and acid. Electrochemical measurements for (a) DMF with bromide salts, and (b) GBL with iodide salts; neat solvent (black diamonds), with 1 M salts (green), with 3 vol% FAH (red), and with 1 M salts and 3 vol% FAH (blue). All potential data has been temperature corrected; temperature is denoted by the background color with the scale bar centered in between the plots.

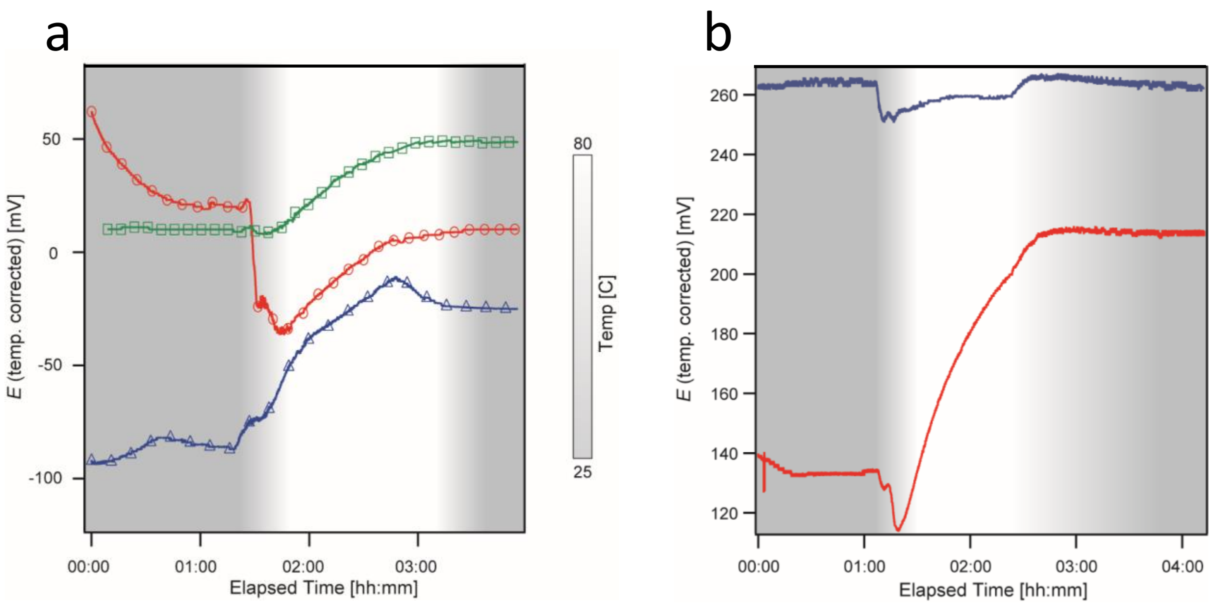

Supplementary Figure 4| Electrochemical data for neat solvents. Potential measurements for neat solvents during heating; all potential data has been temperature corrected with the temperature denoted by the background color with the scale bar centered in between the plots. (a) fresh, anhydrous DMF (red circles), the same sample immediately after the first heating cycle (green squares), and 3 month old DMF (blue triangles); (b) fresh, anhydrous GBL (red), and 1 month old GBL

stored in ambient, after an initial 24 hours at 60 °C, with the septum cap removed (blue). Temperature is denoted by the background color with the scale bar centered in between the plots.

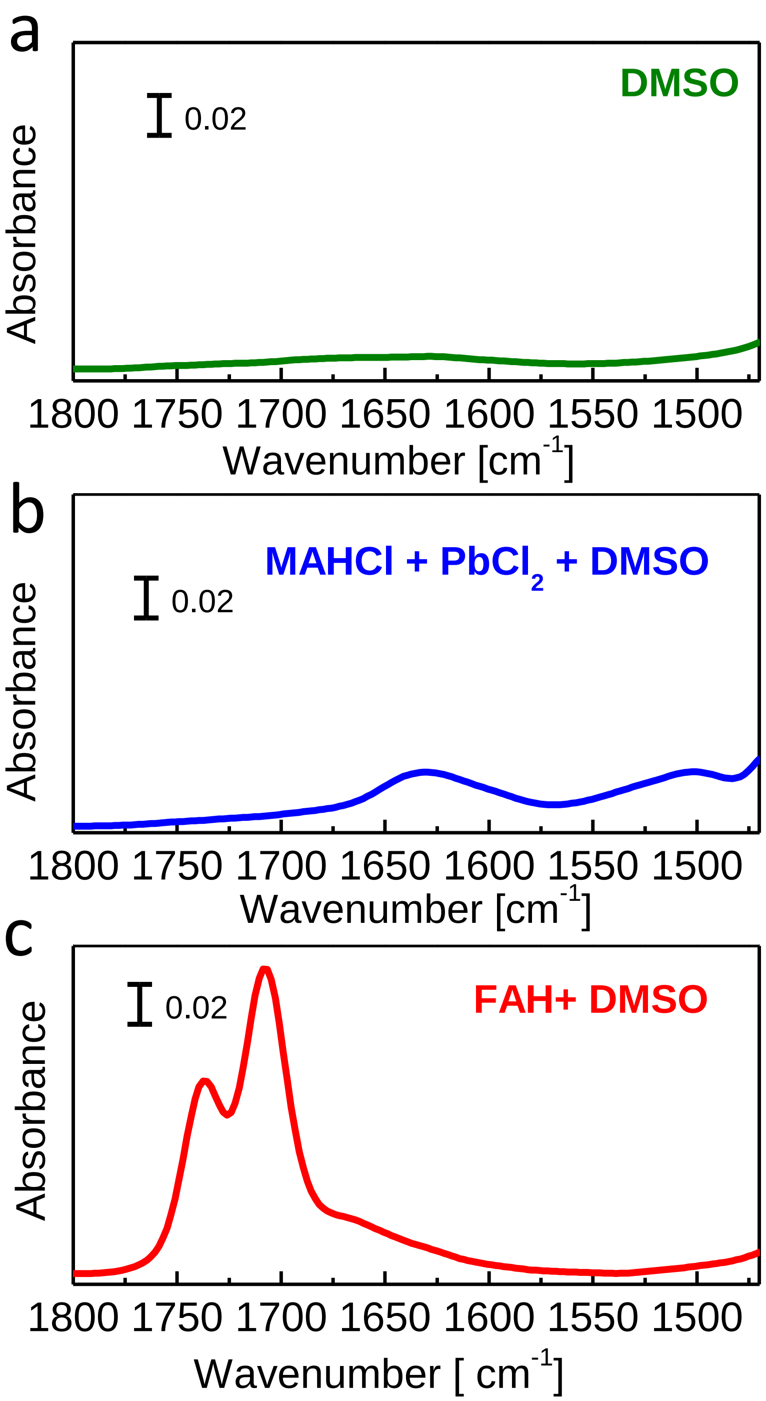

Supplementary Figure 5| FT-IR Controls. (a) IR spectra of neat DMSO, (b) MAHCl + $PbCl_2$ in DMSO and (c) formic acid in DMSO

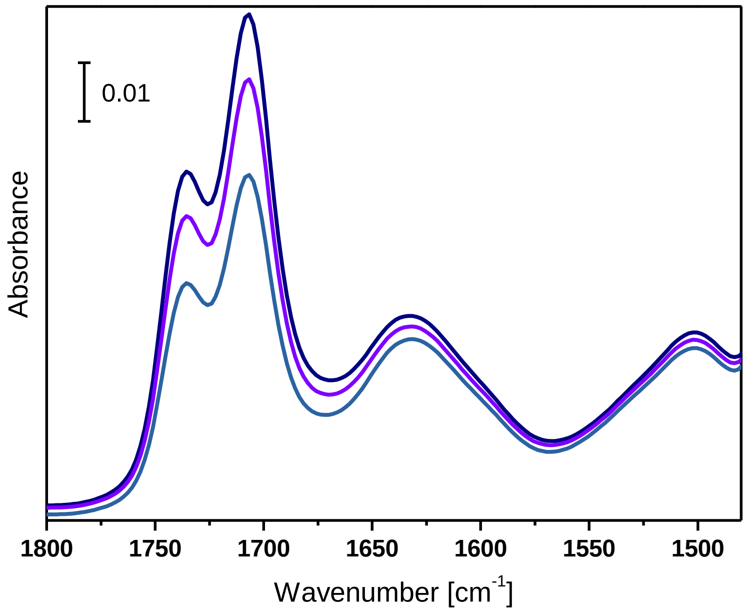

Supplementary Figure 6| IR spectra as a function of acid concentration. IR spectra of MAHCl + $PbCl_2$ in DMSO with different quantity of formic acid.

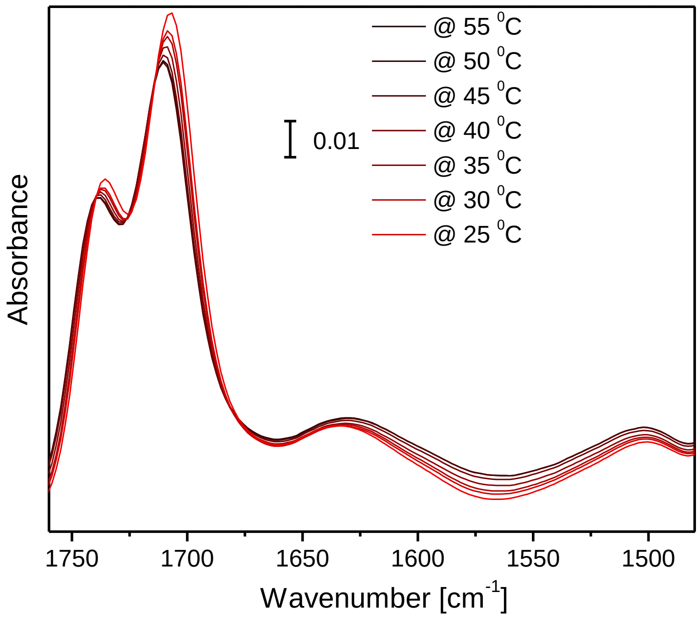

Supplementary Figure 7| IR spectra as a function of temperature. Temperature dependent in situ IR spectra of 1.5 M MAHCl + $PbCl_2$ in DMSO with 5 vol% formic acid.

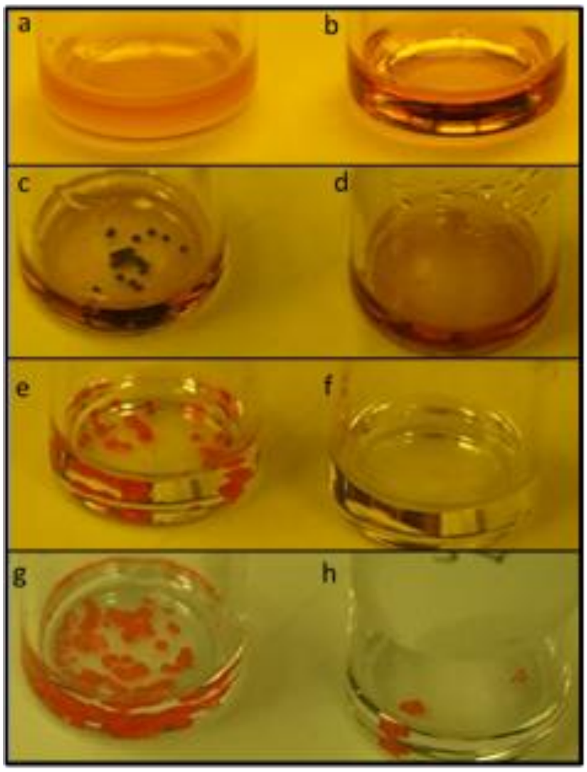

Supplementary Figure 8| Effect of colloids on crystallization. (a) and (b) show the optical images of 1 M salt solution of MAHI +PbI_2 in GBL and supernatant solution after centrifugation, respectively. (c) and (d) show the images when the original and supernatant solution of the iodide salts are incubated at 110°C, respectively. (e) and (f) show the images for 1M MAHBr +$PbBr_2$ solution in DMF and its supernatant solution incubated at 85°C, respectively. (g) and (h) show when the above-mentioned bromide salt solutions are incubated at 120 °C where a higher yield of crystals happens for the original solution.

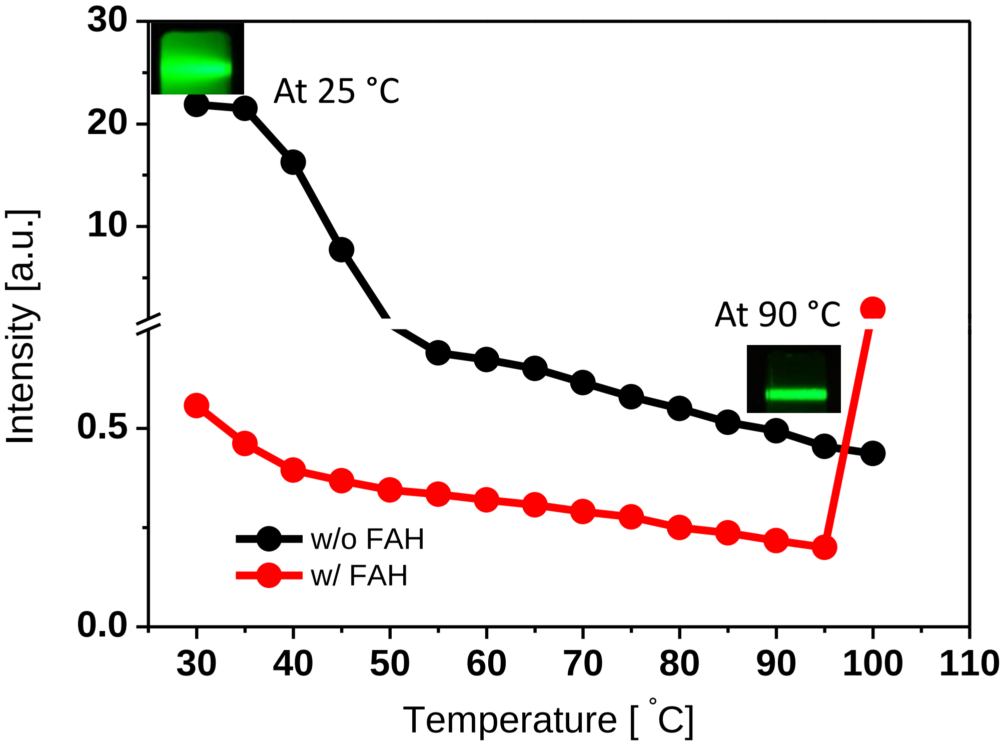

Supplementary Figure 9| SLS Data for iodide system. Effect of added acid and temperature on the scattered light intensity of 1 M MAHI + PbI_2 in GBL, Insets show images of laser beam through 1 M MAHI + PbI_2 at 25°C and at 90°C.

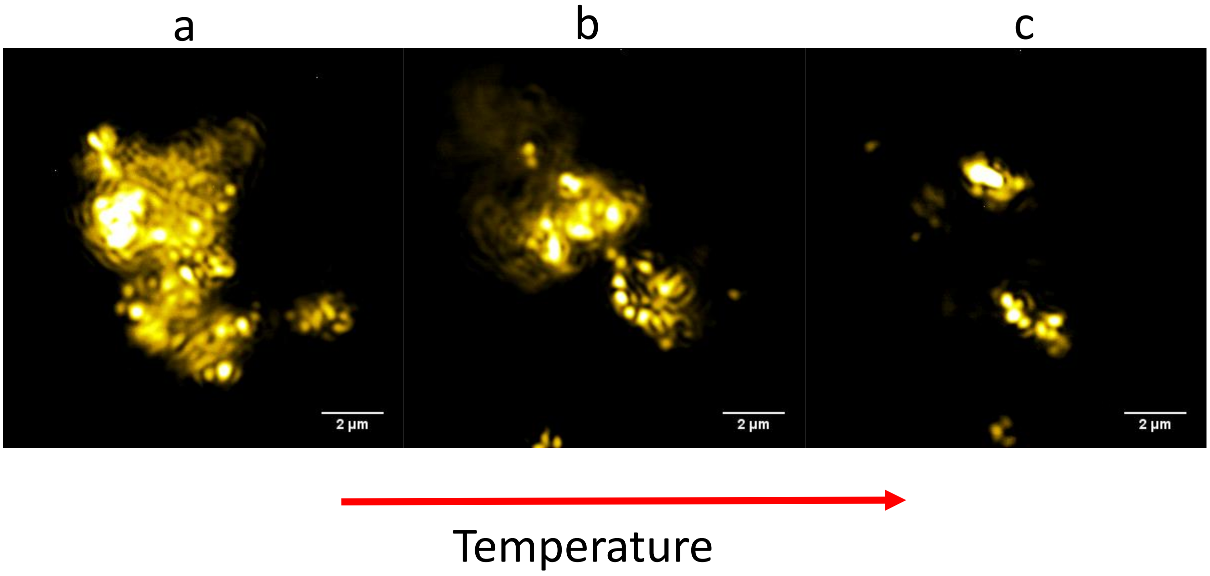

Supplementary Figure 10| iSCAT Data for iodide system. Sequence of events (a-c) during heating of a 1 M iodide salt solution in GBL (temperature range 25°C to 50°C).

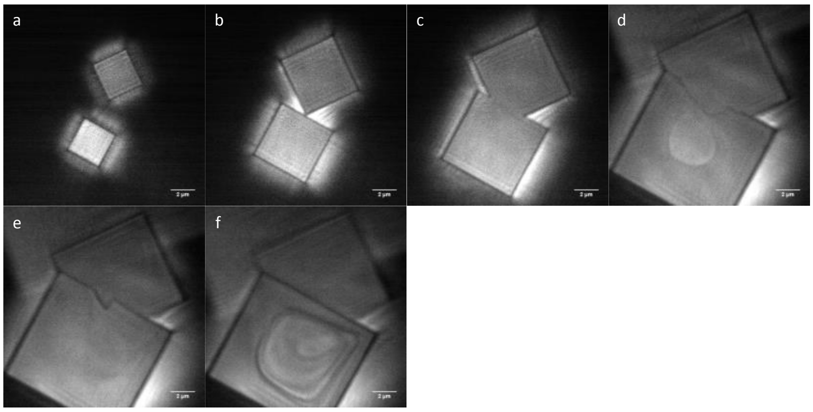

Supplementary Figure 11| Optical images of $CH_3NH_3Br_3$ growth. Sequence of events (a-f) showing the growth of $CH_3NH_3Br_3$ crystal formation where the crystals are growing from clear solution rather than from detectable colloids.

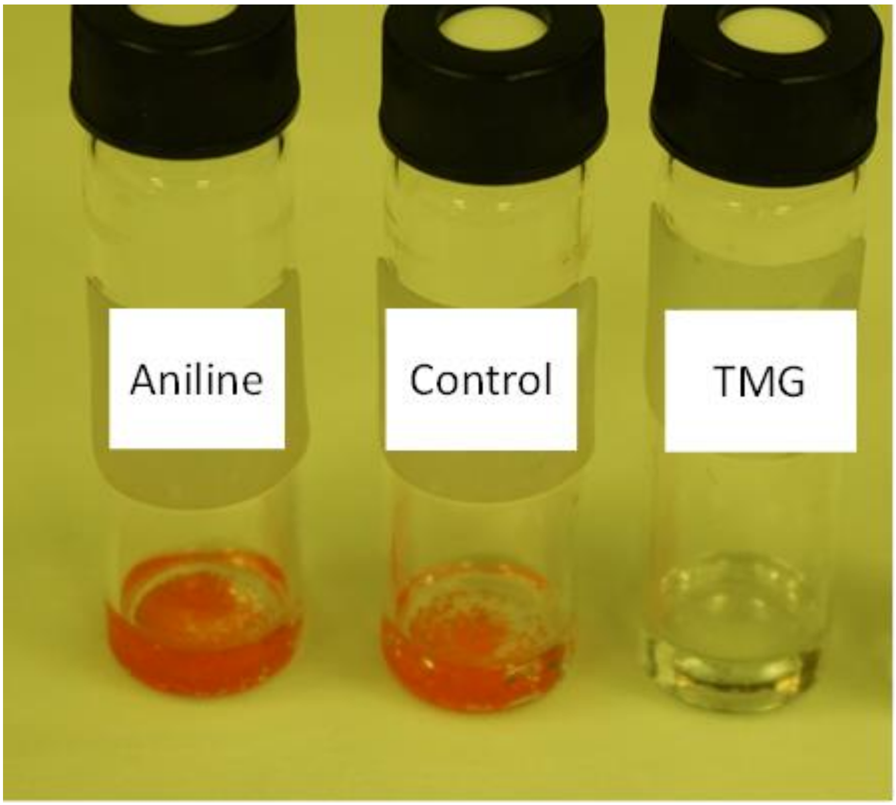

Supplementary Figure 12| Effect of added base. Growth of $CH_3NH_3PbBr_3$ crystals in DMF with weaker / stronger base. A weaker base (Aniline) does not inhibit the crystal formation while a stronger base (TMG) does.

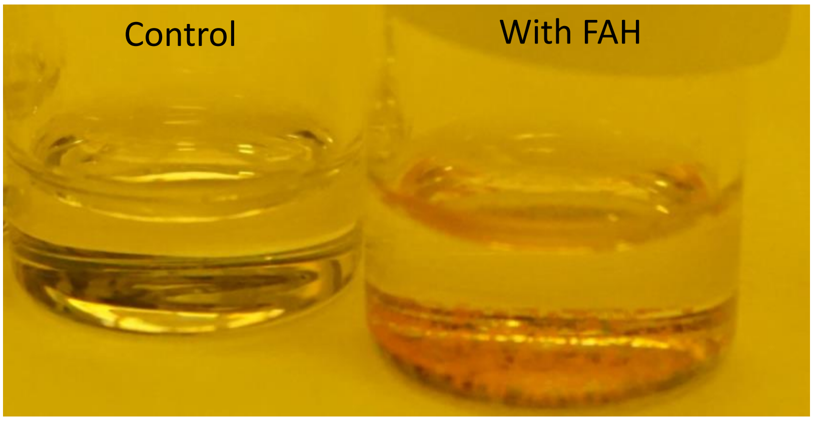

Supplementary Figure 13| Optical image of $CsPbBr_3$ growth. Growth of $CsPbBr_3$ in DMSO with FAH

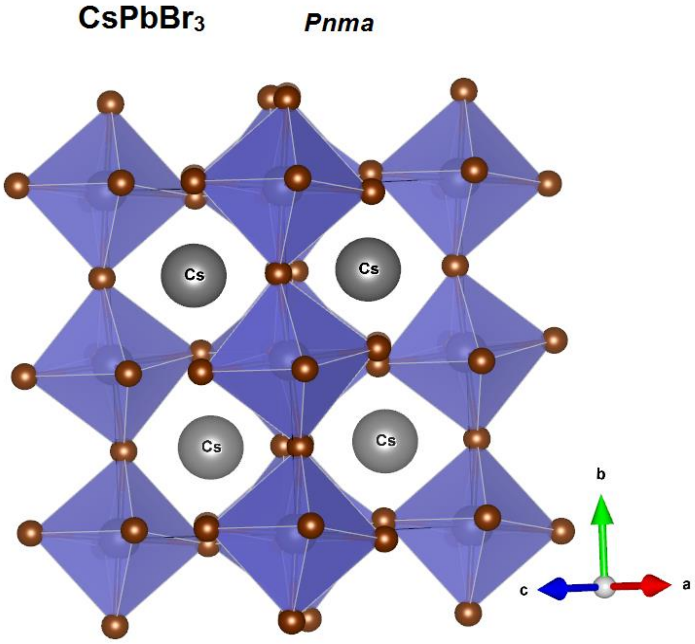

Supplementary Figure 14 | Crystal structure of $CsPbBr_3$. Polyhedral rendering of $CsPbBr_3$ crystal structure; Cs are marked, Br occupy the octahedral vertices with Pb occupying the center of the octahedra.

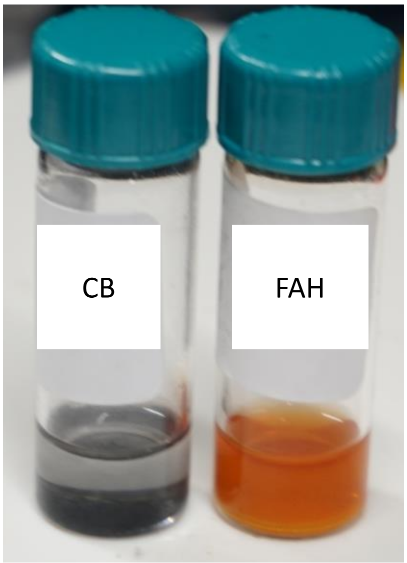

Supplementary Figure 15| Optical image of $MAPbI_3$ powder in CB and FAH. In CB (left) typical anti-solvent behaviour is observed, while FAH (right) acts as a weak solvent.

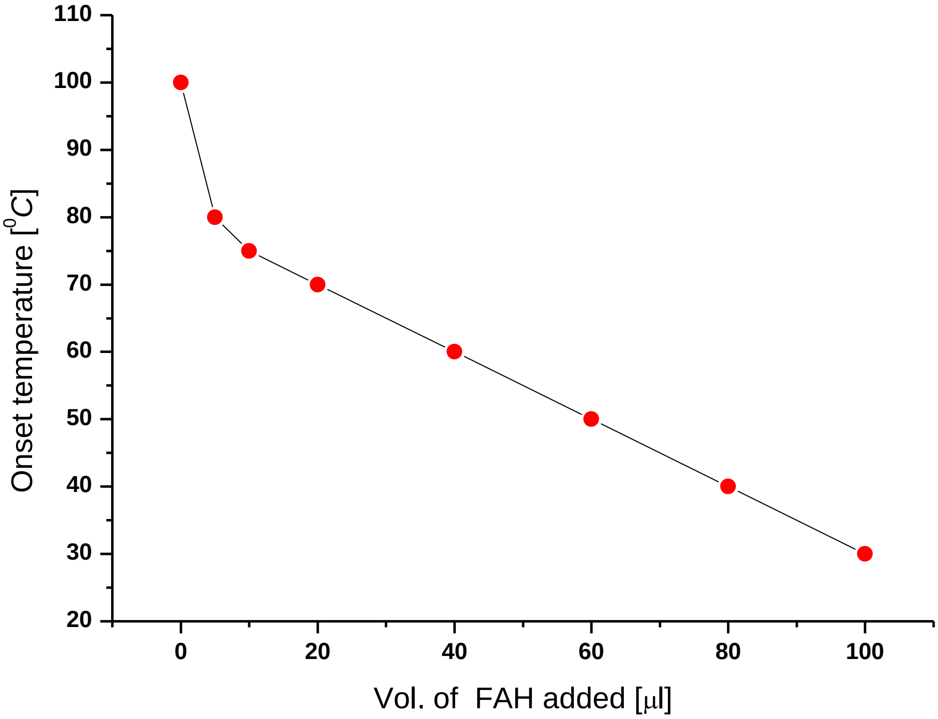

Supplementary Figure 16| Onset temperature as a function of acid concentration. Onset temperature for crystallization of $CH_3NH_3PbBr_3$ versus the volume of FAH added (per ml) in the salt solution.

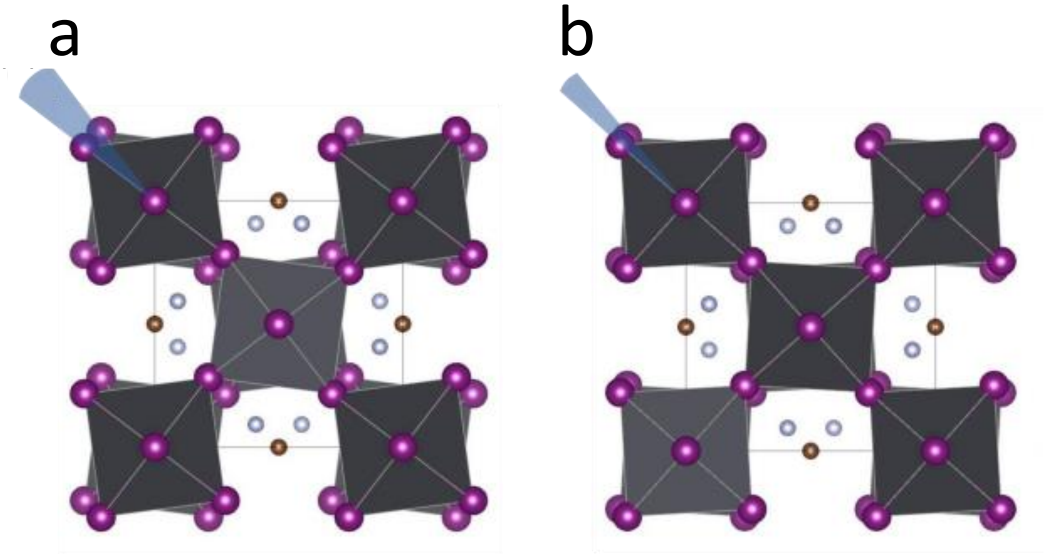

Supplementary Figure 17| Crystallographic data for $CH_3NH_3PbI_3$ as a function of growth temperature. Refined crystal structure of $CH_3NH_3PbI_3$ single crystals viewed along the c-axis grown at a) 55 ˚C and b) 100 ˚C. The octahedral tilting (Glazer tilt i.e. $a^{\circ}a^{\circ}c^{-}$) is illustrated by the dihedral angle (blue area).

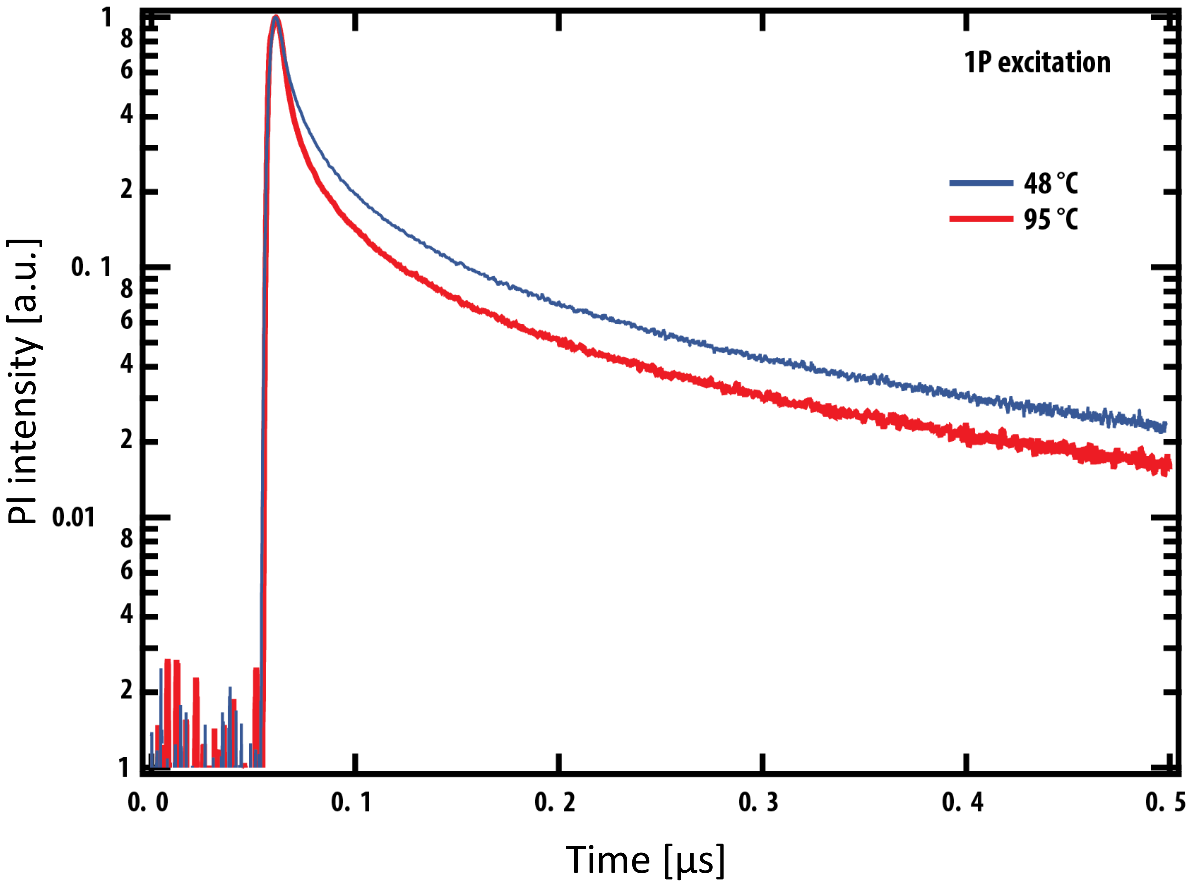

Supplementary Figure 18| 1 Photon TRPL data. Photoluminescent transient of $CH_3NH_3PbI_3$ single crystals grown at 48˚C (below the tetragonal to cubic phase transition) and at 95˚C. under 1P excitation (λ_{ex} = 532 nm).

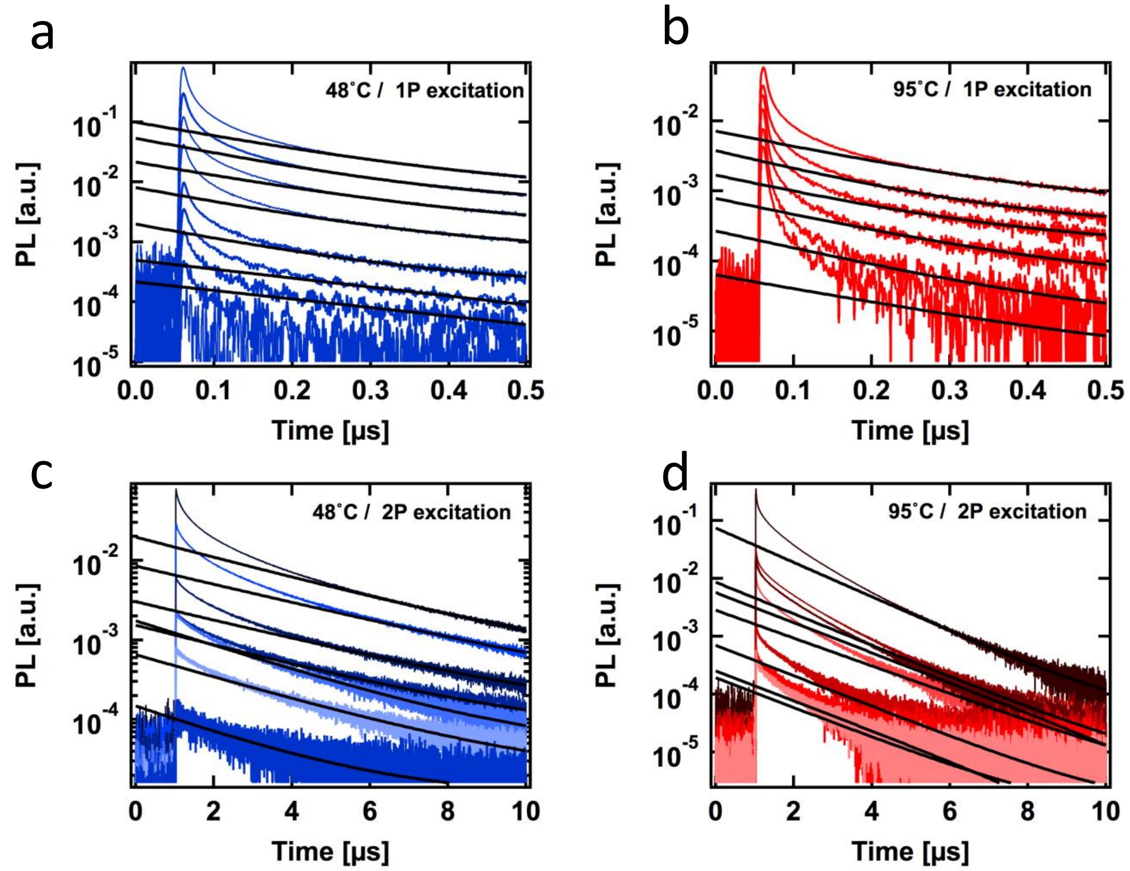

Supplementary Figure 19 | Fluence dependent TRPL data. Decay traces from single crystals grown at 48˚C and 95˚C under 1 photon excitation (a, b) and 2 photon excitation (c, d). The slow part of the decays is fitted with a single exponential to estimate the SRH recombination constant k_1. Excitation fluences for 1 photon excitation at 532 nm: 7.8, 14.3, 30.9, 54.5, 73.3 and 131.8 µJ cm^{-2}; for 2 photon excitation at 1400 nm: 6.3, 9.0, 12.8, 13.2, 17.4, 26.6 and 37.3 mJ cm^{-2}.

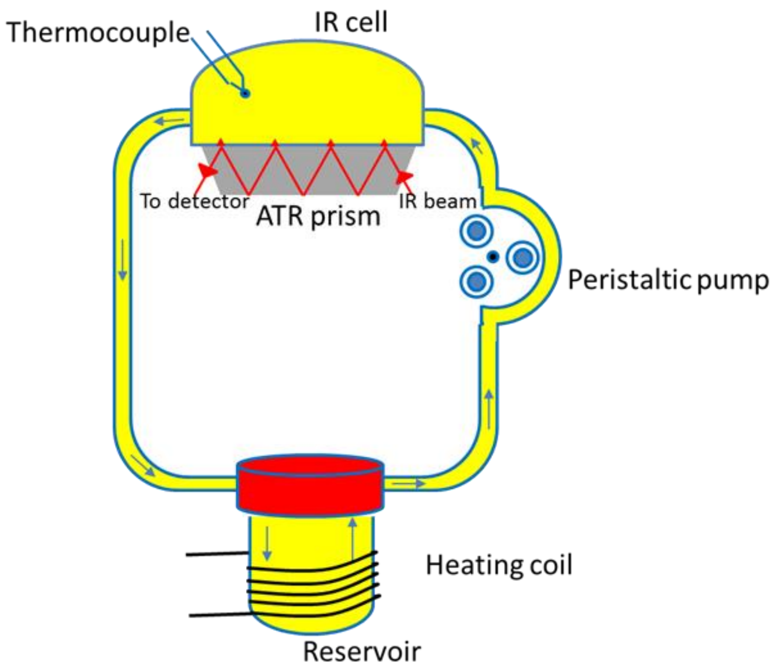

Supplementary Figure 20| Schematic diagram of in situ IR spectroscopy set up. The circulation of solution between the reservoir and the cell is shown by blue arrows.

Supplementary Table 1: Crystallographic data of $CH_3NH_3PbCl_3$,$CH_3NH_3PbBr_3$ and $CH_3NH_3PbI_3$ single crystals at RT.

Compound	$CH_3NH_3PbCl_3$	$CH_3NH_3PbBr_3$	$CH_3NH_3PbI_3$
Growth temperature	328 K	328K	328 K
Measurement temperature	293 K	293 K	293 K
Space group	*Pm-3m*	*Pm-3m*	*I4/m c m*
Unit cell dimensions	a = 5.688 ± 0.005 Å<br>$\alpha = \beta = \gamma$ = 90°	a = 5.928755 ± 0.000078 Å<br>$\alpha = \beta = \gamma$ = 90°	a = 8.878 ± 0.005 Å<br>c = 12.643 ± 0.005 Å<br>$\alpha = \beta = \gamma$ = 90°
Volume	184.03 $Å^3$	208.40 $Å^3$	996.73 $Å^3$
Z	1	1	4
Density (calculated)	3.119 g/cm^3	3.817 g/cm^3	4.131 g/cm^3
Reflections collected	2804	2308	7946
Unique reflections	53	60	261
R(int)	0.1580	0.2554	0.1255
R (sigma)	0.0298	0.0476	0.0278
Goodness-of-fit	0.868	0.518	1.217
Final R indices	0.0383	0.0409	0.0560
Wavelength	0.71073 Å	0.71073 Å	0.71073 Å
Weight scheme for the refinement	Weight = 1 / [$sigma^2(Fo^2)$ + (0.1034 * P $)^2$ + 0.00 * P] where P = (Max (Fo^2, 0) + 2 * Fc^2) / 3	Weight = 1 / [$sigma^2(Fo^2)$ + (0.1184 * P $)^2$ + 34.99 * P] where P = (Max (Fo^2, 0) + 2 * Fc^2) / 3	Weight = 1 / [$sigma^2$ (Fo^2) + (0.0681 * P $)^2$ + 82.13 * P] where P = (Max (Fo^2, 0) + 2 * Fc^2) / 3

Supplementary Table 2: Crystallographic data for a $CsPbBr_3$ single crystal

Compound	$CsPbBr_3$
Measurement temperature	293 K
Crystal system	Orthorhombic
Space group	*P n m a* (no.62)
Unit cell dimensions	*a* = 8.250 ± 0.005 Å, *b*= 11.748 ± 0.005 Å, *c* = 8.205 ± 0.005 Å, $\alpha = \beta = \gamma$ = 90°
Volume	2319.32 Å^3
Z	4
Density (calculated)	4.843 g/cm^3
Reflections collected	10130
Unique reflections	708 from which 0 suppressed
R(int)	0.0853
R (sigma)	0.0411
Goodness-of-fit	1.246
Final R indices (R_{all})	0.0347
wR_{obs}	0.0924
Wavelength	0.71073 Å
Weight scheme for the refinement	Weight = 1 / [sigma^2(Fo^2) + (0.0315 * P)^2 + 0.00 * P] where P = (Max (Fo^2, 0) + 2 * Fc^2) / 3
Atomic Wyckoff-positions	Atom Site *x* *y* *z* site occupancy<br>Cs 4a 0 0.5 0 1<br>Pb 4c -0.47055 0.25 0.00631 1<br>Br1 8d 0.29289 0.47563 0.20664 1<br>Br2 4c 0.00336 0. 75 0.04591 1
Isotropic temperature factors (Å^2)	U_{iso} (CS) 0.08637 ± 0.00065, (Pb) 0.03033 ± 0.00034 , (Br1) 0.07510 ± 0.00070, (Br2) 0.08594 ± 0.00103
Anisotropic temperature factor (Å^2)	$U_{11}(Cs)$ = 0.09940 ± 0.00144, $U_{11}(Pb)$ = 0.03053 ± 0.00051, $U_{11}(Br1)$ = 0.05998 ± 0.00113, $U_{11}(Br2)$ = 0.11628 ± 0.00274<br>U_{22}(Cs) = 0.06517± 0.00129, $U_{22}(Pb)$ = 0.02767 ± 0.00048, $U_{22}(Br1)$ = 0.10608 ± 0.00159, $U_{22}(Br2)$ = 0.02648 ± 0.00133 $U_{33}(Cs)$ = 0.09455 ± 0.00144, $U_{33}(Pb)$ = 0.03279 ± 0.00050, $U_{33}(Br1)$ = 0.05925 ± 0.00103, $U_{33}(Br2)$ = 0.11505 ± 0.00224

Supplementary Table 3: General survey of crystal/processing systems reported using rapid-crystallisation pathway studied here.

Salts	Solvent(s)	Previously Reported Temp. Range [°C]	Current Work Temp Range [°C]
MAI + PbI_2	GBL	90-190	48-150
MABr + $PbBr_2$	DMF	50-110	20-110
MABr + $PbBr_2$	NMP	n/a	80
MABr + $PbBr_2$	DMSO	n/a	105
MACl + $PbCl_2$	DMSO	100	50-80

Supplementary Table 4: Crystallographic data of $CH_3NH_3PbI_3$ single crystals grown at different temperatures

Compound	α-$CH_3NH_3PbI_3$ **(1)**	α-$CH_3NH_3PbI_3$ **(2)**	α-$CH_3NH_3PbI_3$ **(3)**
Growth temperature	328 K	343 K	373 K
Measurement temperature	293 K	293 K	293 K
Space group	*I4/m c m*	*I4/m c m*	*I4/m c m*
Unit cell dimensions	a = 8.878 ± 0.005 Å<br>c = 12.643 ± 0.005 Å<br>$\alpha = \beta = \gamma = 90°$	a = 8.880 ± 0.005 Å<br>c = 12.632 ± 0.005 Å<br>$\alpha = \beta = \gamma = 90°$	a = 8.916 ± 0.005 Å<br>c = 12.545 ± 0.005 Å<br>$\alpha = \beta = \gamma = 90°$
Volume	996.73 $Å^3$	996.08 $Å^3$	997.26 $Å^3$
Z	4	4	4
Density (calculated)	4.131 g/cm^3	4.134 g/cm^3	4.132 g/cm^3
Reflections collected	7946	5937	6131
Unique reflections	261	257	260
Inconsistent equivalents	10	18	8
R(int)	0.1255	0.1212	0.0881
R (sigma)	0.0278	0.0265	0.0245
Goodness-of-fit	1.217	2.094	1.419
Final R indices	0.0560	0.0771	0.1269
Twin model	[0 1 0 1 0 0 0 0 -1]	More than one twin	More than one twin
Extinction coefficient	0.028696	0.005836	0.008768
Wavelength	0.71073 Å	0.71073 Å	0.71073 Å
Weight scheme for the refinement	Weight = 1 / [$sigma^2(Fo^2)$ + (0.0681 * P $)^2$ + 82.13 * P] where P = (Max (Fo^2, 0) + 2 * Fc^2) / 3	Weight = 1 / [$sigma^2(Fo^2)$ + (0.0681 * P $)^2$ + 82.13 * P] where P = (Max (Fo^2, 0) + 2 * Fc^2) / 3	Weight = 1 / [$sigma^2(Fo^2)$ + (0.0529 * P $)^2$ + 642.71 * P] where P = (Max (Fo^2, 0) + 2 * Fc^2) / 3

Supplementary Discussion

In situ pH measurement of GBL and DMF

Supplementary Figure 3 shows the erratic behaviour of the solvent with only acid added which is similar to the behaviour of the neat solvents in Supplementary Figure 4. For GBL and DMF this is to be expected as they already contain some concentration of carboxylic acid. Therefore, addition of a similar acid only increases or decreases this behaviour (depending on the solvent age) but it is still erratic and not completely reversible. Despite this behaviour with the solvent/acid samples, for the complete system: solvent, acid, and salts; the change in acidity is of similar magnitude and completely reversible for all systems tested.

In situ ATR-IR measurement

We chose the chloride salt system in DMSO as neat DMSO does not produce any carboxylic acid as degradation product, does not show any change in the proton activity upon heating and has no IR absorption peak in the 1800-1450 cm^{-1} region where we expect to see the carbonyl (from carboxylic acid) and N-H (from MA and MAH^+) vibrational modes. Supplementary Figure 5 shows the ATR-IR absorption spectra of neat DMSO, DMSO containing MAHCl and $PbCl_2$ and DMSO containing FAH. The absorption peaks at 1500 cm^{-1} and 1625 cm^{-1} are due to the symmetric and asymmetric bending modes of N-H in MA and MAH^+, respectively. The intensity of these peaks should reflect the degree of protonation of MA due to the change in symmetry (Cs for MA and C3v for MAH^+) .[1] For carboxylic acids, absorbance around ~1750-1700 cm^{-1} is due to the carbonyl stretching and its intensity decreases up on deprotonation.[2] Supplementary Figure 6 shows IR spectrum of MAHCl + $PbCl_2$ in DMSO with different amount of FAH. We see that addition of more FAH causes an increase in the absorbance of the IR modes associated with carbonyl stretching as well as N-H bending.

In Supplementary Figure 7 we show the temperature dependent IR absorbance of 1.5 M MAHCl + $PbCl_2$ in DMSO with 5 vol % FAH at 5 °C intervals from 25 °C to 55 °C. We see a monotonic decrease in absorbance for the carbonyl stretching vibrations (~1700-1750 cm^{-1}) and monotonic increase for the N-H vibrations (at 1500 cm^{-1} and 1620 cm^{-1}) as the temperature rises. We attribute this to the deprotonation of FAH and protonation of MA on heating.

Test of MA on solvent strength

To understand the effect of MA on the solution strength, we prepare a 1M solution of MABr + $PbBr_2$ in DMF and incubate the solution at 100°C to produce single crystals of $CH_3NH_3PbBr_3$. After 2 hours of incubation, when there is no noticeable further growth of the crystals, we assume that the crystals are in equilibrium with the solution. Then we bubble MA gas through the solution for 5 minutes, while maintaining the crystallisation temperature, and find that the crystals dissolve back into the solvent.

Effect of partial removal of colloids from the solution:

To test the effect of colloids in crystallization, we prepare 1 M solution of bromide salts in DMF and iodide salts in GBL. We stir the iodide salt solution at 70°C for 30 minutes before bringing it to the room temperature. We then centrifuge the solutions at 14800 rpm for 8 minutes. After centrifugation, we collect the supernatant solutions, depleted in colloidal particle concentration. In Supplementary Figure 8 (a) and (b) we show the optical images of 1 M solution of MAHI + PbI_2 in GBL, and supernatant solution after centrifugation. We see that the supernatant solution is visibly clearer than the original solution which we attribute to the partial removal of colloidal particles. When we raise the temperature of the solutions, $MAPbI_3$

crystals start to form at ~110 °C in the original solution (Supplementary Figure 8c) unlike the supernatant solutions where we do not observe any crystal formation until 150 °C (Supplementary Figure 8d). Similarly for the $MAPbBr_3$ system, when we raise the temperature to 85 °C we see the crystal formation in the original solution (Supplementary Figure 8e) unlike its less colloid containing counterpart where the onset of crystallization is at 120 °C (Supplementary Figure 8f). As we show in Supplementary Figure 8 (g) and (h), the yield of the crystals is less in the supernatant solution compared to the original solution under similar conditions.

<u>Hypothesis Tests</u>

<u>Effect of acid/base strength</u>

We test our proposed mechanism by intentionally disrupting the acid/base equilibria and observing the impact on the crystallisation in a solution of 1 M bromide salts in DMF. When we add a weaker base, aniline (protonated base pKa=3.8 in DMSO), than MA (protonated base pKa=11.0 in DMSO) to the solution we expect no change in the crystallisation behaviour as the MA will be preferentially protonated during heating.[3] When we add a stronger base, 1,1,3,3-Tetramethylguanidine (TMG) (protonated base pKa=13.2 in DMSO), we expect the stronger base to be protonated, in lieu of MA, which should inhibit the crystallisation. In both test cases the behaviour we observe matches our expectations (Supplementary Figure 12).[3]

Finally, we crystallise the iodide system in GBL isothermally at 70 ºC by addition of FAH; once the crystals have formed we add a slightly stronger base to the solution (butylamine, protonated base pKa=11.1 in DMSO) and the crystals dissolve; i.e. we crystallise by addition of acid and reverse it by addition of base.[3]

$CsPbBr_3$ crystals:

We prepared 0.33 M solution of $PbBr_2$ and CsBr in DMSO and added 20 vol % of formic acid. By heating the resulting solution at 120 °C for 10 minutes, we produced single crystals of $CsPbBr_3$. XRD single crystal diffraction spectra confirmed the formation of $CsPbBr_3$ crystals; see Supplementary Figures 13 and 14 and Supplementary Table 2.

Test of FAH as an anti-solvent:

To find out whether FAH is an anti-solvent for perovskites like chlorobenzene (CB), we added 5 mg of $MAPbI_3$ powder (prepared by crushing single crystals of $MAPbI_3$ in a mortar and pestle) to vials containing 1ml of FAH and CB. We then stirred the solution for 30 minutes. We then measure photoluminescence (PL) of the solutions. While the CB solution showed the PL corresponding to the $MAPbI_3$, the FAH solution did not show the same behaviour. Supplementary Figure 15 shows the optical image of the vials containing CB and FAH solution containing $MAPbI_3$ powders. In FAH solution, $MAPbI_3$ powder does not survive in its perovskite form, thus FAH is not a typical antisolvent like CB.

Isothermal Growth

To verify that this crystallisation effect is completely independent of temperature we crystallise the iodide, bromide and chloride systems isothermally at 70, 55, and 70 °C respectively. Details are the same as given in the Method sections in the main text with the following differences: (1) the solutions were dissolved at the crystallisation temperature and filtered through a 0.2 um syringe filter into two vials (1 control and 1 for acid addition), (2) the acid was preheated in the same oil bath for ~30 minutes before addition, and (3) vials

were removed from the bath long enough to add the acid to the solution and agitate by hand until visible cloudiness was removed (typically 1-2 minutes). Results for the iodide and bromide system are shown in Figure 1 in the main text.

Measurement of onset of crystallization

We prepared 1 M solution of the bromide salts in DMF. We then used a closed cap vial with the solution placed in an oil bath to monitor the onset of temperature of crystallization as a function of addition of formic acid (FAH). The temperature range was 20-100 °C and the step size was 10 °C .We waited 5 minutes at each temperature so see whether crystallization has occurred or not by visual inspection. Once we saw crystallization, we noted the temperature and allowed the bath to come to 20 °C (ambient temperature) and the crystals to dissolve back in the solution. We then added few µls of FAH to vial and repeat the experiment to note the onset temperature. The experiment was repeated to see the effect of additional formic acid on the onset temperature of crystallization. Supplementary Figure 16 shows the correlation between added FAH and onset temperature for crystallization.

Measurement of yield for crystallization:

We prepared 1 M solution of the bromide salts in 10 ml of DMF. We then used two closed cap vials each with half of the salt solution to grow the crystals. To one of the vials we added 100 µl of formic acid (FAH) and then placed both in an oil bath at 90 °C to grow crystals. After 30 minutes, we collected the $CH_3NH_3PbBr_3$ crystals, followed by a washing with dry diethyl ether and drying in an oven at 65 °C for 10 minutes. We then weighed the crystals to calculate the yield. We found that the yield of the crystals formation was 45% from the vial with FAH, compared to 35% from the control. We then brought the vials to room temperature and again

added another 100µl of FAH to the vial already with FAH, then put both the vials again in the oil bath for another 30 minutes. We did not notice any growth of crystals in the control vial whereas the vial with FAH yielded more crystals which were collected as described before. We repeated the experiment with further addition of FAH followed by collection of crystals. We found that while the control vial did not yield more crystals after the initial incubation, incremental addition of FAH can give a cumulative yield up to 85%.

Crystallographic data of $CH_3NH_3PbI_3$ grown at different temperatures:

The diffraction patterns from the crystals grown at 55 °C are fit with only a single twin, and they exhibit the best fit against the calculated diffraction pattern. Another key observation is that the crystal structure we refine from the crystals grown at differing temperatures have significant differences. For a cubic perovskite lattice, we expect there to be no rotational tilt between the adjacent lead halide octahedra, for a tetragonal lattice we expect this tilt to exist. Interestingly, the tilt angle between adjacent octahedra is larger for the crystals grown at 55 °C than those grown at 100 °C, exemplifying the importance of controlling the temperature of the crystallisation in order to control the crystal structure, and hence the resulting electronic properties.

Supplementary References:

1. Zeroka, D. & Jensen, J. O. Infrared spectra of some isotopomers of methylamine and the methylammonium ion: a theoretical study. *J. Mol. Struct. THEOCHEM* **425,** 181–192 (1998).
2. Max, J.-J. & Chapados, C. Infrared Spectroscopy of Aqueous Carboxylic Acids: Comparison between Different Acids and Their Salts. *J. Phys. Chem. A* **108,** 3324–3337 (2004).
3. Cox, B. G. *Acids and Bases*. **16,** (Oxford University Press, 2013).
